# Prevalence of primary open angle glaucoma in the last 20 years: a meta-analysis and systematic review

**DOI:** 10.1038/s41598-021-92971-w

**Published:** 2021-07-02

**Authors:** Nan Zhang, Jiaxing Wang, Ying Li, Bing Jiang

**Affiliations:** 1 Department of Ophthalmology, The Second Xiangya Hospital, Central South University, Changsha, 410011 Hunan China; 2 Department of Ophthalmology, School of Medicine, Emory University, Atlanta, GA USA; 3 Hunan Clinical Research Center of Ophthalmic Disease, Changsha, China

**Keywords:** Ocular hypertension, Vision disorders, Risk factors

## Abstract

Primary open-angle glaucoma (POAG) is a leading cause of irreversible blindness in the world and is influenced by various sociodemographic factors. This meta-analysis aims to determine the worldwide prevalence of POAG in the adult general population for the last 20 years, and explore variation in prevalence by age, gender and geographical location. An electronic literature search was performed using the PubMed, Embase, and Web of Science databases. Population-based cross-sectional or cohort studies published in the last 20 years (2000–2020) that reported prevalence of POAG were recruited. Relevant studies meeting defined eligibility criteria were selected and reviewed systematically by meta-analysis. The prevalence of POAG was analyzed according to various risk factors. A random effect model was used for the meta-analysis. Fifty publications with a total of 198,259 subjects were included in this meta-analysis. The worldwide overall prevalence of POAG was 2.4% (95% CI 2.0 ~ 2.8%). The prevalence increases with age. Men are found to be more susceptible to POAG than women (RR 1.28, p < 0.01). Africa is found to have the highest prevalence of POAG (4.0%) among all continents. The current estimated global population of POAG is 68.56 million (95% CI 59.99 ~ 79.98). POAG is a worldwide vision threatening disease with high prevalence for the last 20 years. The population-based prevalence of POAG varies widely across individual studies, due to variations in risk factors of age, gender, and population geographic location.

## Introduction

Glaucoma is the leading cause of irreversible blindness and second leading cause of blindness, leading to a huge burden of the world^1,2^. Primary open angle glaucoma (POAG) is the predominant subtype of glaucoma. The number of POAG cases in adult population (40–80 years old) was estimated 52.68 million in 2020 and 79.76 million in 2040^3^. The population-based prevalence of POAG varies widely across individual studies, due to variations in risk factors such as age, gender, and population geographic location^4,5^.

Efforts has been made to estimate the regional or global POAG prevalence using meta-analysis^3,6,7^, in which they covered epidemiological studies performed from 1960 to 2014. It was reported that the prevalence from older and historic studies were significantly different from surveys after year of 2000 due to updates in studies designs and diagnostic methods^7^. Moreover, there has been numerous epidemiological studies about POAG published in the past few years, with large number of worldwide participants. Therefore, in this meta-analysis, we estimated the global prevalence of POAG for the recent 20 years (2000–2020) in a risk factor specific manner.

## Methods

The study was conducted following the Preferred Reporting Items for guidelines of Systematic Reviews and Meta-analysis (PRISMA) guidelines^8,9^.

### Eligibility criteria

All population-based cross-sectional or cohort studies published from January 2000 to October 2020 that had prevalence of POAG or could calculated by available data were collected and included in this meta-analysis. Inclusion criteria include: (1) Cohort or cross-sectional studies; (2) Population-based study of POAG from a defined geographic region; (3) Clear definition of random or clustered sampling procedure; (4) Studies that prevalence data for POAG can be extracted or calculated; (5) POAG defined by using International Society of Geographic and Epidemiologic Ophthalmology (ISGEO) criteria^10^ or similar to ISGEO that definition based on evidence for structural or functional features of glaucomatous optic neuropathy, independent of intraocular pressure (IOP).

Exclusion criteria include: (1) Studies were not general population-based (e.g. hospital-based or clinical based); (2) Studies without number of subjects with glaucoma; (3) Self-reported diagnosis of glaucoma included; (4) Non-English articles; (5) Articles using repeated data from the author’s previous publications.

### Search strategy

Systematic and comprehensive search were performed on August 2020 in several electronic databases (PubMed, Embase and Web of Science) to identify publications. Publications from January 2000 to October 2020 were searched using search strategy combined the keywords “primary open-angle glaucoma”, “POAG” with “prevalence”, “population”, “survey”, “epidemiology”. To acquire studies comprehensively, we also conducted a manual search or reference lists from targeted articles. Detailed search strategy of different databases was listed in Supplementary Table S1.

### Risk of bias assessment

Articles included in the study were assessed for risk of bias using 2 domains of the Quality in Prognosis Studies tool (QUIPS)^11^ that are relevant to observational studies (study participation and outcome measurement)^12^. Appraisal of each domain provides a subjective assessment of risk of bias (ranked as low, moderate, or high). A summary of the areas considered in the assessment of each domain is included in Supplementary Tables S2 and S3.

### Data extraction

NZ (primary reviewer) and YL (secondary reviewer) independently screened the titles and abstracts for each paper found in the search procedure and obtained full-text versions of all potentially eligible studies. Once full-text publications had been retrieved, the reviewers checked the studies again and applied eligibility criteria to further exclude papers. All disagreements received final consensus after several full discussions between reviewers. Full data extraction in the data extraction sheet was completed after reviewers independently identified cases from every targeted article and reached final agreement. Data of first author, year of publication, gender, age, continent (we classified regions according to the United Nations’ classification of continental regions^13^: Africa, Asia, Europe, North America, South America (Latin America and the Caribbean), Oceania), country, habitation area (urban or rural), numbers of cases, sample size, and prevalence with 95% confidence interval (CI), response rate were extracted and reported for each study in Supplementary Table S4.

### Statistical analysis

Data are presented as prevalence (95% CIs). Forest plots were performed using the software R version 3.6.3 (R Foundation for Statistical Computing, Vienna, Austria) and the R package “meta”^14^. We selected the prevalence of POAG as the main outcome. The relative risk ratios (RRs) and 95% CIs of the results were compared. Heterogeneity between studies was quantified using I^2^ statistic^15–18^. Due to the high likelihood of heterogeneity among the selected studies, we used a random effects model to evaluate pooled effects. Publication bias was calculated using the contour-enhanced funnel plot^19,20^, P-curve analysis^21^, and Egger test^19^ (p < 0.05 was considered as significant publication bias). Detailed bias for each study were described in Supplementary Table S2 and S3.

The p-value for prevalence difference among groups for age, continent, habitation area, and decades were calculated using “metaprop” from R package “meta”, random effects model. The p-value for prevalence difference among groups for gender were calculated using “metabin” from R package “meta”, random effects model. Meta-regression test was performed for subgroup analysis, with the first category of each subgroup used as intercept. The statistical output includes a test of whether the intercept differs significantly from zero, and whether other groups differs with the intercept. A value of p < 0.05 was considered statistically significant. We obtained the population projection from the latest data of the World Population Prospects of the United Nations^22^ and our random effects model. The estimated numbers of POAG population were calculated by the age- and region-specific prevalence and the corresponding total population number.

## Results

### Search results

**Figure 1:**
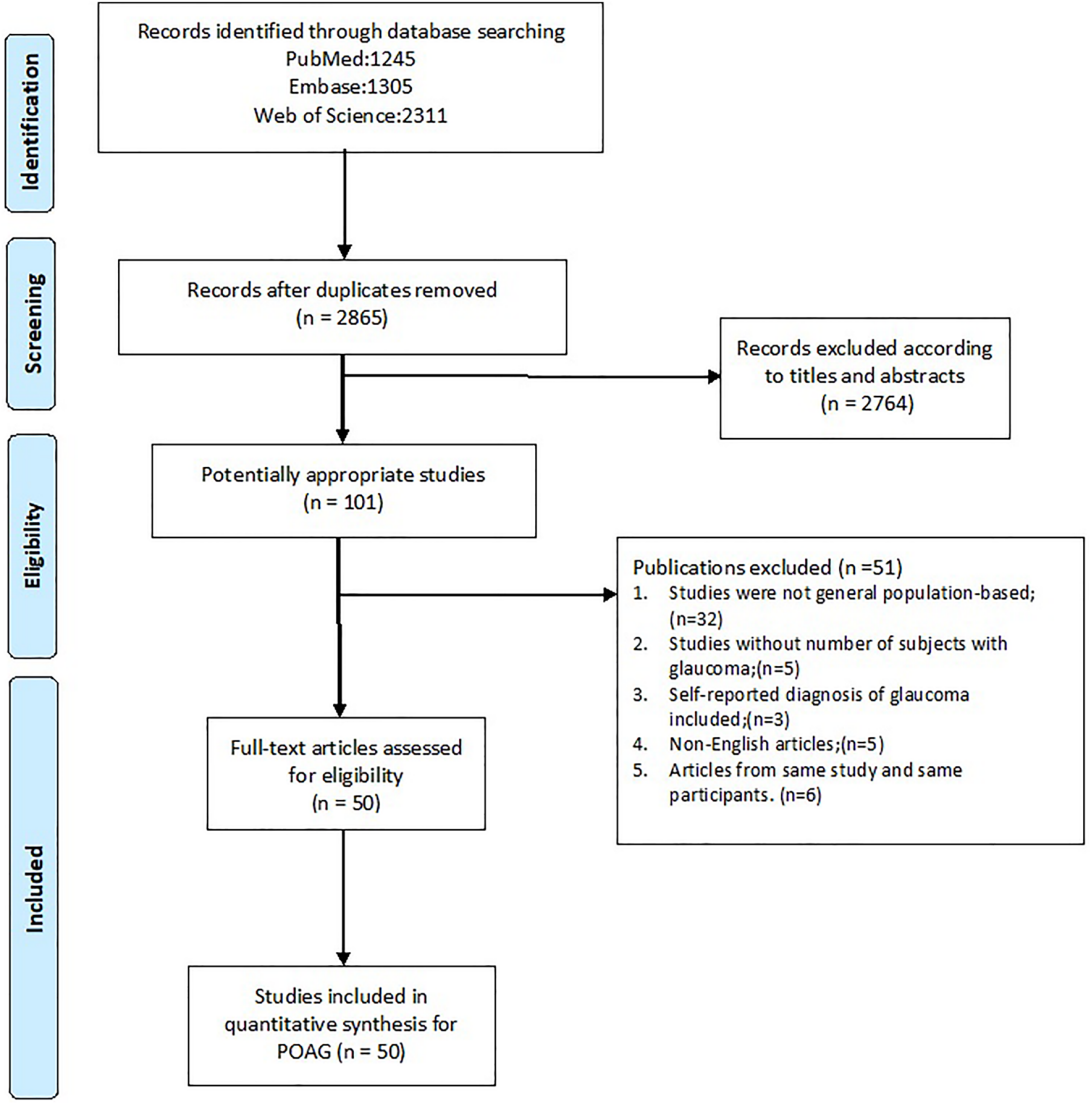
Flow charts of search strategy. The flow chart illustrates the search strategy following the Preferred Reporting Items for guidelines of Systematic Reviews and Meta-analysis (PRISMA) guidelines. *POAG* primary open-angle glaucoma.

In this study, we systematic reviewed all population-based studies about POAG prevalence which were published in the last 20 years. The search returned a total of 2865 publications, leading to 101 articles selected for full-text review. A total of 50 articles^23–72^ had their full-text reviewed for inclusion. The screening process is detailed in Fig. 1. A total of 50 publications that include 198,259 subjects were finally selected for inclusion in the review. The sample size of study ranged from 569 (Anton, 2004)^34^ to 15,122 (Chassid, 2018)^70^. Detailed information including author, year of publication, country, continent, age range, response rate, detailed number of cases and sample size are provided in forest plots given different risk factors and summarized in Supplementary Table S4.

### Risk of bias

A summary of the risk of bias of the included articles is provided in supplementary materials (Supplementary Figs. S1 and S2); a justification of each rating is provided in the Supplementary Tables S2 and S3. For potential bias derived from selection of study participants, 28 studies (56%) were considered to be at low risk and 22 studies (44%) were considered to be at moderate risk. One study^43^ was considered at high risk for the reason that they have included only 73 + years of age subjects in their study, which overestimated the prevalence for general population.

For potential bias derived from outcome measurement, 32 (64%) studies were considered to be at low risk, 12 (24%) studies were considered to be at moderate risk for not using ISGEO 2002 criteria but described in details of their own criteria that similar to ISGEO 2002. Six (12%) studies^28,31,38,43,51,53^ reported open-angle glaucoma (OAG) instead of POAG, which may cause overestimation of the prevalence of POAG, and therefore considered to be at high risk of bias for outcome measurement. For overall risk of bias, 20 studies (40%) were considered to be at low risk of bias, 22 studies (44%) were considered to be at moderate risk of bias, and 8 (16%) studies were considered to be at high risk of bias.

Egger test result revealed a significant publication bias (p < 0.01) in this meta-analysis. Funnel plots and P-curve analysis results were shown in Supplementary Figs. S3 and S4.

### Prevalence of POAG

**Figure 2:**
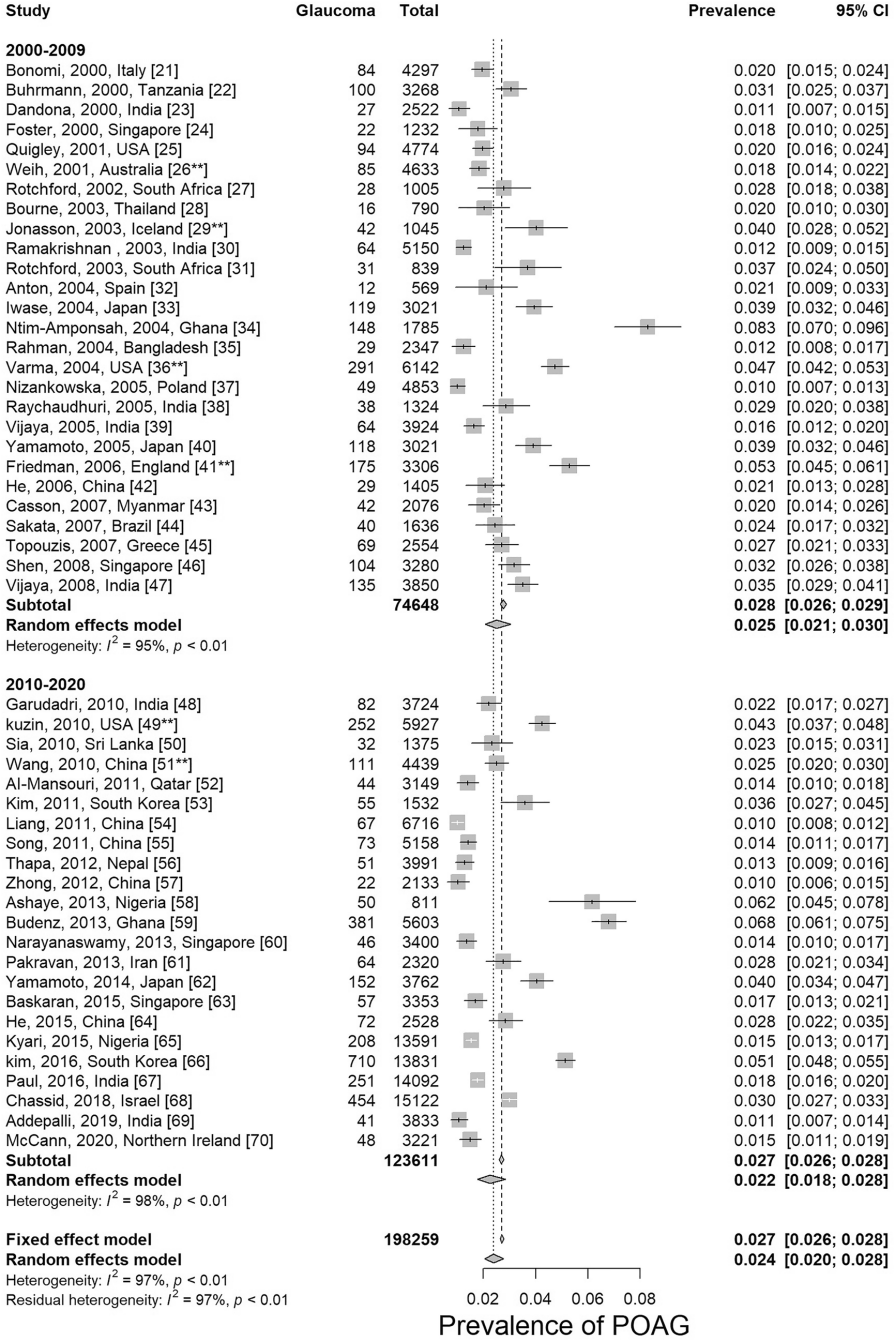
Prevalence of primary open-angle glaucoma by decades. *POAG* primary open-angle glaucoma. There is no statistical difference between the first decade (2000–2009) and the second decade (2010–2020) in the global prevalence of POAG (p = 0.472). **Studies that reported prevalence of open-angle glaucoma (OAG), but not POAG.

The prevalence data for POAG are summarized in Table 1. The overall prevalence of POAG worldwide is 2.4% (95% CI 2.0% ~ 2.8%) for the last 20 years (Fig. 2). When compare the first (2000–2009) with the second decade (2010–2020), we found no change in the global prevalence of POAG (Q = 0.52, df = 1, p = 0.472, random effective model). Note that the prevalence reported in 6 of the studies^28,31,38,43,51,53^ (indicated by ** in all forest plots) are for open-angle glaucoma (OAG), but not POAG. Therefore, the prevalence data from these 6 studies were slightly overestimated for POAG.

**Table 1:** Results of subgroup analyses and meta-regression analyses based on age, gender, geographical location, habitation area, decades and risk of bias.

	Subgroup analysis				Meta-regression	
	Number of estimates	Pooled estimate (95% CIs)	I^2^, %	p-value (between groups)	β coefficient value (95% CIs)	p-value
All estimates	**50**	2.4 (2.1 to 2.8)	96.8			
**Age range**				< 0.01	Intercept = “ < 40”	
< 40	3	0.4 (0 to 4.4)	92.2		− 5.01 (− 5.93 to − 4.09)	< 0.01
40–49	23	1.1 (0.8 to 1.7)	94.4		0.57 (− 0.40 to 1.54)	0.25
50–59	26	2.0 (1.6 to 2.6)	91.2		1.11 (0.15 to 2.07)	0.02
60–69	26	3.3 (2.6 to 4.1)	92.0		1.62 (0.66 to 2.58)	< 0.01
70–79	19	6.0 (4.7 to 7.6)	89.7		2.25 (1.28 to 3.22)	< 0.01
70 +	8	4.4 (2.9 to 6.8)	88.5		1.94 (0.88 to 2.99)	< 0.01
80 +	18	9.2 (7.1 to 11.9)	73.9		2.68 (1.69 to 3.66)	< 0.01
**Gender**				< 0.01	Intercept = Male	
Male	33	2.7 (2.2 to 3.2)	93.7		− 3.60 (− 3.80 to − 3.41)	< 0.01
Female	33	2.0 (1.7 to 2.5)	93.9		− 0.27 (− 0.55 to 0.01)	< 0.01
**Geographical location**				< 0.01	Intercept = Africa	
Africa	7	4.0 (2.6 to 6.1)	97.4		− 3.17 (− 3.54 to − 2.80)	< 0.01
Asia	31	2.1 (1.8 to 2.4)	95.2		− 0.69 (− 1.09 to − 0.28)	< 0.01
Europe	7	2.3 (1.5 to 3.5)	94.6		− 0.57 (− 1.10 to − 0.04)	0.03
N. America	3	2.4 (1.8 to 3.3)	96.6		− 0.17 (− 0.83 to 0.50)	0.63
Oceania	1	3.4 (2.2 to 5.3)	–		− 0.81 (− 1.85 to 0.22)	0.12
S. America	1	1.8 (1.5 to 2.3)	–		− 0.53 (− 1.59 to 0.53)	0.33
**Habitation area**				0.09	Intercept = Urban	
Urban	14	2.5 (1.9 to 3.2)	94.4		− 3.67 (− 3.95 to − 3.40)	< 0.01
Rural	16	1.9 (1.5 to 2.3)	91.3		− 0.28 (− 0.65 to 0.09)	0.14
Mixed or unknown	26	2.3 (2.0 to 2.7)	97.5		0.05 (− 0.29 to 0.38)	0.79
**Decades**				0.47	Intercept = 2000–2009	
2000–2009	27	2.5 (2.1 to 3.1)	94.8		− 3.66 (− 3.87 to − 3.45)	< 0.01
2010–2019	23	2.2 (1.8 to 2.8)	97.7		− 0.11 (− 0.42 to 0.20)	0.48
**Risk of bias**				0.01	Intercept = Low	
Low	20	2.5 (2.0 to 3.2)	96.3		− 3.64 (− 3.87 to − 3.41)	< 0.01
Moderate	24	2.0 (1.6 to 2.5)	95.9		− 0.24 (− 0.55 to 0.08)	0.14
High	6	3.5 (2.6 to 4.8)	95.3		0.34 (− 0.13 to 0.81)	0.16

*S. America* South America, *N. America* North America.

### Gender variation

**Figure 3:**
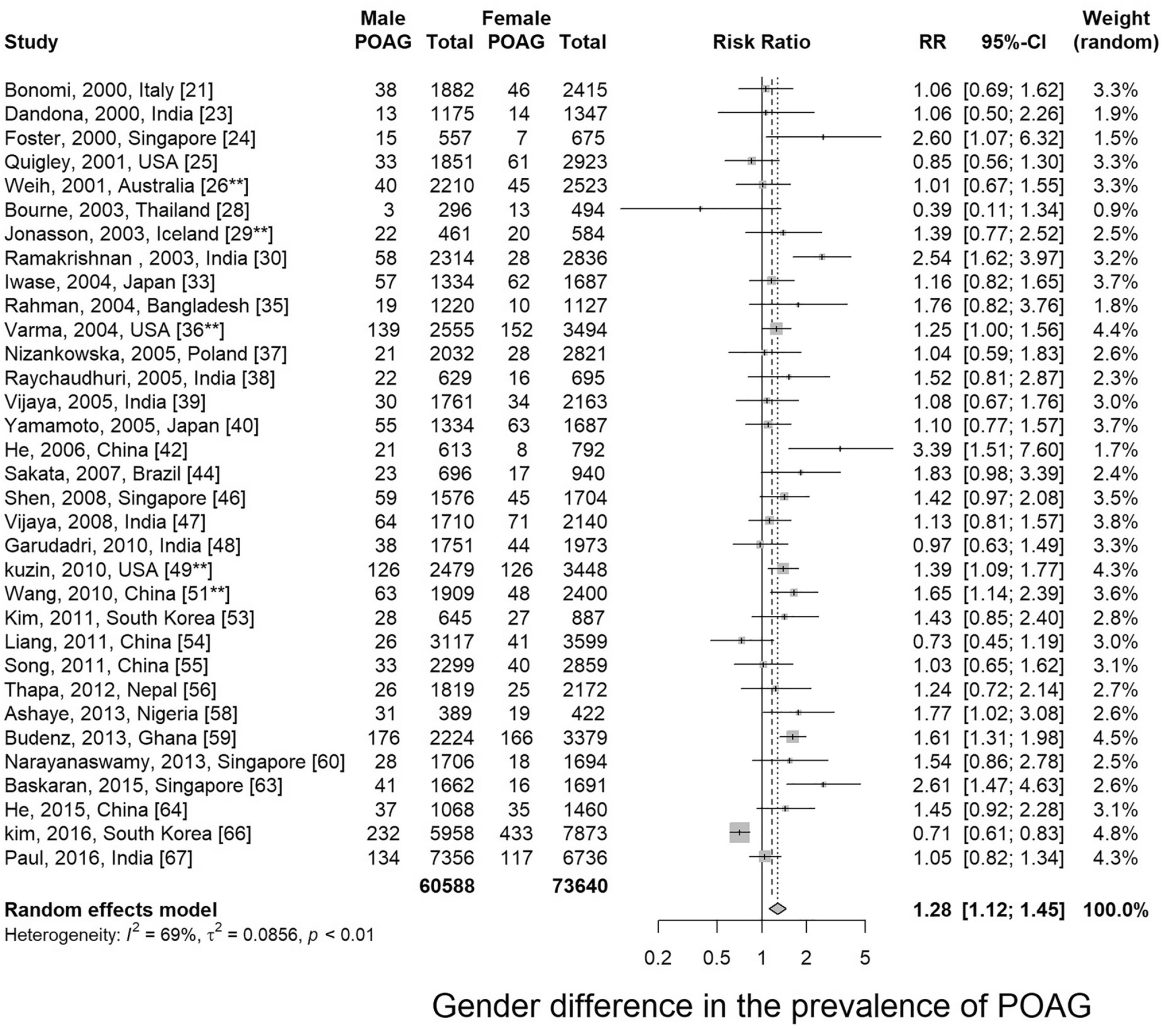
Gender comparison of primary open-angle glaucoma. *POAG* primary open-angle glaucoma. **Studies that reported prevalence of open-angle glaucoma (OAG), but not POAG.

Thirty-two articles presented prevalence data by gender. Prevalence was higher for men in 84.4% of the studies (27 out of 32). Male-to-female prevalence ratios ranged from 0.39 to 2.61. Meta-analysis showed that men are more susceptible to POAG than women with a relative risk of 1.28 (95% CI 1.12 ~ 1.45, p < 0.01) (Fig. 3 and Supplementary Fig. S5).

### Age variation

**Figure 4:**
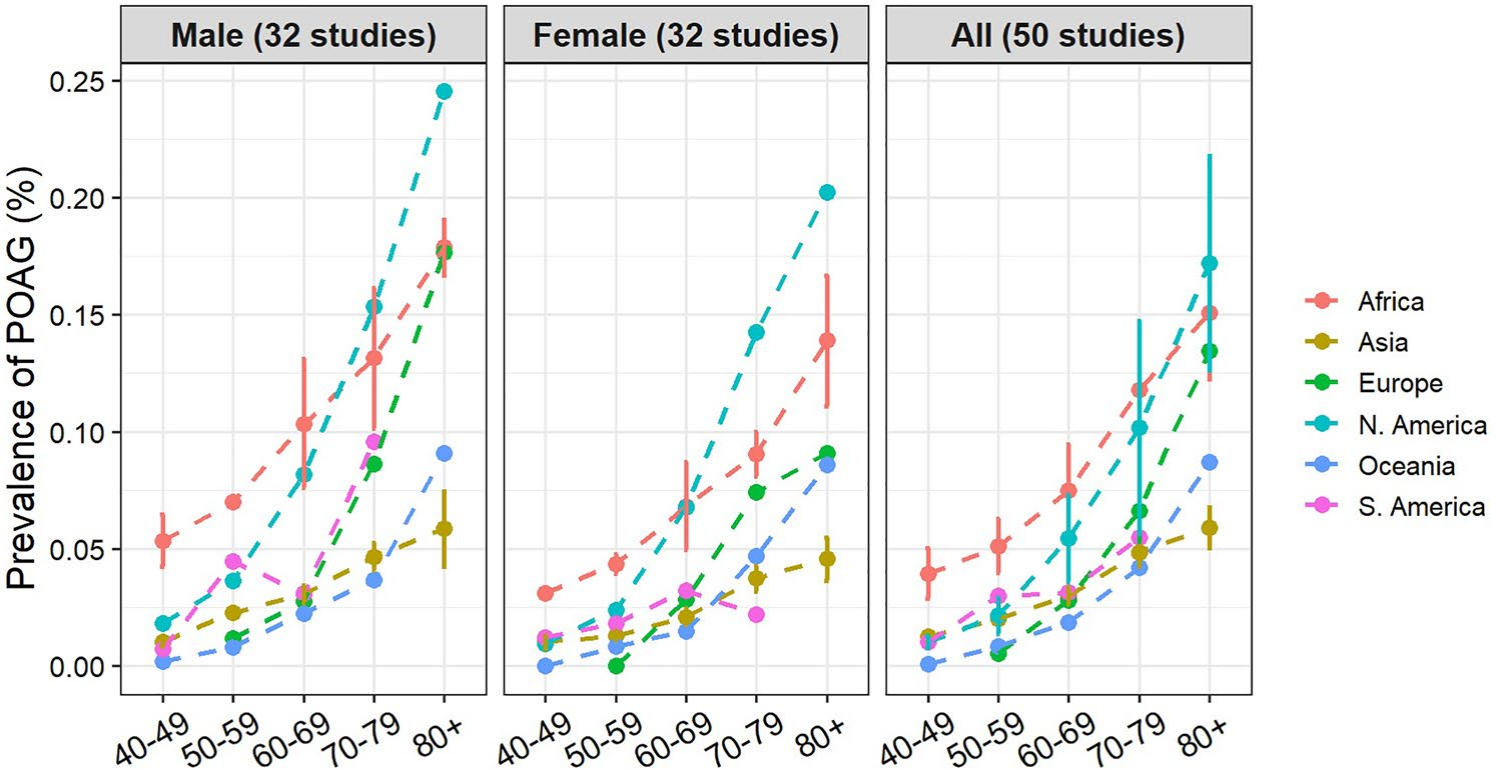
Prevalence of primary open-angle glaucoma for different age groups. Note: the plots for “Male” and “Female” represent studies with data for each gender (n = 32). The plot for “Both” represents data from all studies (n = 50), regardless of whether the prevalence for each gender were reported or not. *POAG* primary open-angle glaucoma.

Thirty-nine studies (78%) recruited participants with age of 40+, 8 studies (16%) recruited participants with age of 50+, and one study^43^ recruited participants with only age of 73 and over. Thirty-seven articles presented prevalence data by age groups. Age-specific prevalence of POAG is presented in Fig. 4 by continents and genders. The prevalence of POAG increase with age, ranging from 1.1% (0.8 ~ 1.7%) at age of 40 ~ 49 to 9.2% (7.0 ~ 12.1%) at age over 80 (Table 1, Supplementary Fig. S6). Subgroup differences tested using random effects model revealed a statistically significant difference among different age groups in the prevalence of POAG (Q = 122.90, df = 6, p < 0.001, random effective model).

### Geographical variation

**Figure 5:**
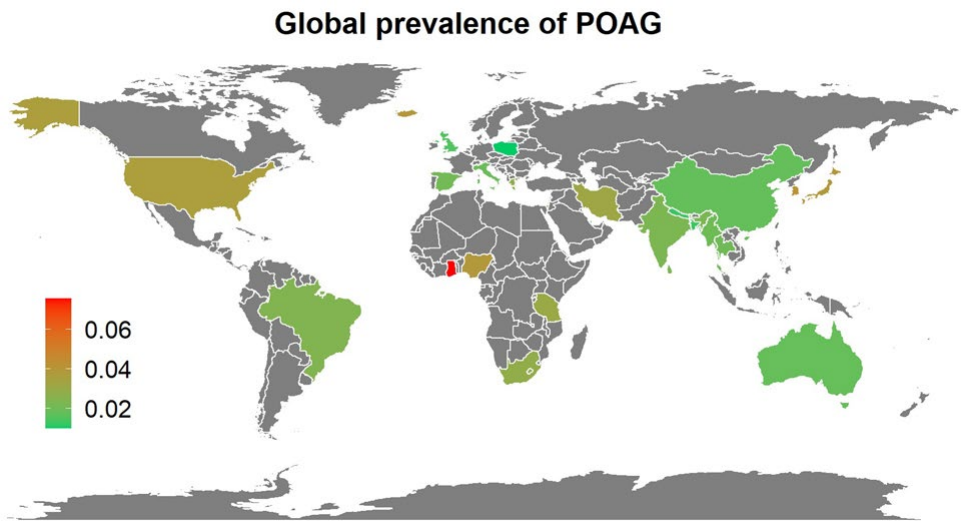
Global prevalence of primary open-angle glaucoma. This world map was created using R (version 3.6.3) and R package “ggplot2”. The country codes (ISO3) used in the program were downloaded from www.nationsonline.org.

Prevalence of POAG for each continent were summarized in Table 1 and Fig. 5. Among all continents, Africa (data from Tanzania, South Africa, Ghana and Nigeria) is found to have the highest prevalence of POAG 4.0% (2.6 ~ 6.1%) and Oceania (data from Australia) is found to have the lowest prevalence 1.8% (1.5 ~ 2.3%) (Supplementary Fig. S7). Subgroup differences tested using random effects model revealed a statistically significant difference among different continents in the prevalence of POAG (Q = 15.65, df = 5, p < 0.001).

### Habitation area variation

The information of habitation area (urban or rural) is reported in 26 studies. The prevalence for rural and urban are listed in Table 1 as well as Supplementary Fig. S8. Subgroup differences tested using random effects model revealed a p-value of 0.089 (Q = 4.82, df = 2), indicating no statistical difference among different habitation areas.

### Heterogeneity and meta-regression analysis

The overall random-effects pooled prevalence of POAG was 2.4% (95% Cis 2.0 ~ 2.8%) with a high level of heterogeneity (I^2^ = 96.8%). When only studies at low risk of bias (on both domains of the Quality in Prognosis Studies tool) were considered, the pooled prevalence remains at 2.5% (95% Cis 2.0 ~ 3.2%), with unchanged heterogeneity of 96.3% (Table 1, Supplementary Fig. S9). Meta-analysis stratified by risk of overall bias was performed and the result showed no significant change in the heterogeneity among the three risk levels (Table 1). However, the estimated prevalence is increased in the high risk group and is higher than the low and moderate risk groups (3.5% vs 2.5% and 2.0%, Table 1), representing the overestimation of prevalence majorly due to the involvement of other types of OAG in the high risk studies. The heterogeneity (I^2^) drops when the estimates were sub-grouped by age and gender (Table 1). The most significant drop of heterogeneity is patients of over 80 years old (I^2^ = 73.9%). These findings indicate that the population variations, gender and age is the major contributory factors to heterogeneity.

The results of meta-regression analyses including pooled estimates for subgroups based on age, gender, geographical location, habitation area, decades and risk of bias are included in Table 1. There was little evidence of an effect of habitation area, decades or risk of bias on prevalence. However, there were apparent higher prevalence in males, older aged people and the continent of Africa.

### Estimation of current global burden of POAG

According to the World Population Prospects of the United Nations^22^, the current global population is about 7794.8 million, population over 40 years old is about 2856.5 million. Based on the results of this meta-analysis, the estimated total population of POAG in the world is about 68.56 million in 2020 and over 53% of them are in Asia. The detailed estimation of glaucoma population in each continent are listed in Table 2.

**Table 2:** Estimated global population of glaucoma.

Continent	Population over 40 years old in 2020 (million)	Estimated POAG cases (million, 95% CI)	Distribution of POAG cases (continent/world)
Africa	266.91	10.68 (6.94–16.28)	15.58%
Asia	1756.85	36.89 (31.62–43.92)	53.81%
Europe	400.34	9.21 (6.01–14.01)	13.43%
S. America	162.7	3.90 (2.93–5.40)	5.69%
N. America	177.68	6.04 (3.91–9.42)	8.81%
Oceania	17.38	0.31 (0.26–0.40)	0.45%
World	2856.52	68.56 (59.99–79.98)	

*POAG* primary open-angle glaucoma, *S. America* South America, *N. America* North America.

## Discussion

This study provided the most updated worldwide prevalence of POAG for the last 20 years. Based on our results, the overall global prevalence of POAG on population over 40 years old is 2.4% (95% CI 2.0% ~ 2.8%). This estimation is very similar with Kapetanakis et al.’s report (2.3%) in 2016^7^, and slightly lower than the estimation from Tham et al.’s report (3.05%) in 2013^3^. It is not comforting that the global prevalence of POAG hasn’t been dropped in the last decade (2010–2020) when compared with 2000–2009 based on our results (Table 1, Fig. 2). In this study, we estimate the current global population of POAG is about 68.56 million, slightly higher than the estimated number from Kapetanakis et.al (65.46 million)^7^ and Tham et.al (52.68 million)^3^. POAG is still a worldwide public health burden that requires improvement in diagnostic and therapeutic approaches, particularly in populations with high prevalence. Such population can be identified by the risk factors including age, gender and ethnicity or geographic locations.

### Age

Age is known to be the major risk factor for POAG, as the prevalence increase as people get older^73,74^. This is confirmed in this meta-analysis. Population over 80 years has highest risk have POAG (9.2%) among all age groups. Aging per decades consistently associated with higher IOP, thinner central corneal thickness, which are the major contributions to higher prevalence of POAG in aged population^75^.

### Gender

In this study, male gender is found to be a significant risk factor for POAG (RR 1.28, 95% CI 1.12 ~ 1.45, p < 0.01), in consistent with other reports^4,76^. The exact reasons remain unclear. Multiple studies have suggested men had longer axial length and deeper anterior chamber depth (ACD)^77–79^. In contrast with POAG, female is one of the risk factors of PACG^80^. Such anatomical differences could be potential reasons of the gender difference we observed in POAG.

### Race and geographical locations

Race is another known risk factor for POAG. Vajaranant, TS et al. estimated that Hispanics/Latinos will be the largest group in POAG patients as compared with other ethnicities^5^. Friedman et al. reported that POAG prevalence of black subjects was almost 3 times than white subjects (OR 2.82; 95% CI 2.14–3.72)^81^, which is consistent with our findings that Africa has the highest prevalence of POAG. These findings were also supported by other studies^3,7^, which reported prevalence of POAG is 4.20% (2.08%; 7.35%) and 5.2% (3.7%; 7.2%) for African people, respectively. Africans are reported to have smaller trabecular meshwork height which might diminished outflow facility^82^. In addition, thinner central corneal thickness was also reported to be associated with African, which might be another contributor to the development of POAG^83,84^. The other possible reason could be the higher environmental temperature the Africans originally lived. It is reported that the prevalence of POAG for people at age of 70+ increased with average monthly maximum temperature^85^.

In this meta-analysis, majority of the included studies (30 of 50) were conducted in Asia countries. While majority of the ethnicity from Asia countries are Asian, people from other countries like the USA and Europe were with mixed ethnicity. Since most of the studies are lack of detailed prevalence data for each ethnicity, the power of meta-analysis sub-grouped by ethnicity is very limited. Hence, geographical location differences were analyzed in this study instead. Among the continents, Africa is found to have the highest prevalence of POAG (4.0%), while Oceania had the lowest (1.8%). North America ranked second highest POAG prevalence in our study (3.4%). The prevalence might be overestimated since we only included 3 studies from North America and two of them are studies of OAG prevalence. The number of epidemiological investigations of glaucoma in North America in the past two decades is limited. In 2004, Friedman et al. published a meta-analysis about POAG prevalence and related risk factors of USA, in which they included studies from 1985 to 2000^81^. In their study, the overall prevalence of POAG was 1.86% (95% CI 1.75–1.96%) for population over 40 years old in USA and they estimate that the number of POAG patients will be 3.36 million in 2020. Another meta-analysis reported that there’s 2.71 million POAG patients in 2011 and the number will reach to 7.32 million in 2050 in the US.

Asia accounts about 60% of global glaucoma population^6^. We estimated 36.89 million (95% CI 31.62 ~ 43.92 million) POAG cases in Asia in 2020. More efforts in screening and treatment of POAG should be considered in Asia. The prevalence of POAG also varies in different Asia regions. South-central Asia was considered to have highest burden of POAG and overall glaucoma than other regions, while the East Asia is reported to have higher prevalence of angle closure glaucoma^6^.

### Habitation area

Besides gender, age and continents, habitation area (urban or rural) were also analyzed in this study (Supplementary Fig. S8). We found no statistical difference among different habitation areas (p = 0.089). However, this part of analysis represents substantial bias for the following reasons: (1) The information habitation area is usually vaguely described in majority of the studies; (2) There are only few studies that have included both urban and rural populations in the study, and therefore the comparison between urban and rural across studies represent ethnicity and country bias. For POAG, there are only 2 studies that have compared both settings and they conclude different. In Weih et al. study in 2001 on Australian population^28^, the prevalence of POAG is higher in rural (2.12%) than urban area (1.72%). However, in Paul et al. study of India population in 2016^69^, they found the prevalence of POAG is higher in urban (2.10%) than rural area (1.45%). A meta-analysis based on Chinese population also showed that people living in urban area are more likely to have POAG [OR 1.54 (95% CI 1.02 ~ 2.35)]^86^. However, since our meta-analysis represent bias for above reasons, more evidence is needed to reveal the role of habitation area in the risk of POAG in future studies.

### Bias and heterogeneity

The risk of bias in this meta-analysis were from the following four major aspects: selection of participants, response rate, diagnosis criteria and involvement of other types of OAG (Supplementary Figs. S1, S2). The overall risk of bias is low for the reason that low quality studies were excluded as mentioned in the method. Beyond that, a meta-regression for studies at different level of risk of bias was performed, showing that the risk of bias for included studies plays no major role in the overall bias of this meta-analysis.

In this meta-analysis, the overall heterogeneity is high (I^2^ = 96.8%). It is common that a meta-analysis for prevalence studies yields very high heterogeneities, that the I^2^ value are usually over 90%^12,87–90^. The heterogeneity did not drop when only studies at low risk of bias were analyzed, but dropped dramatically in the subgroup population of over 80 years old (I^2^ = 73.9%) (Table 1). It suggests that the heterogeneity in pooled prevalence estimates is due to the risk factors of disease such as age, gender, geographical location of the studies and the variations among studies. Given the high heterogeneity among studies, the pooled prevalence estimate should therefore be interpreted with caution.

### Limitations

The first limitation of this study is that the number of studies conducted in last 20 years varies a lot across continents, and therefore the overall prevalence for some continents represent selection bias. There are only 1 study for South America^46^, 1 studies for Oceania^28^ and 3 studies in North America^27,38,51^. Second, there are 6 studies that investigated OAG, instead of just POAG in our analysis, including 1 from Asia^53^, 1 from Oceania^28^, 2 from Europe^31,43^ and 2 from North America^38,51^, and therefore the overall prevalence of POAG in this meta-analysis is slightly over estimated, especially for those continents. However, since POAG is the major contribution of the overall prevalence for OAG, and the number of studies in these continents are very limited, the value of these studies outweighs their risks and therefore were included in this meta-analysis. Third, the diagnostic criteria of POAG differs among studies, even though the majority of our included studies used same ISGEO criteria. Besides, the ISGEO criteria is almost 20 years old and disadvantages were known of this definition. The bias from the POAG diagnostic criteria across studies should not be ignored.

## Conclusion

In this meta-analysis, we reviewed 50 studies of 198,259 subjects for the prevalence of glaucoma in the last 20 years. Detailed prevalence according to different age, gender, continents, countries and habitation area was reported. Up to date, POAG is still a worldwide vision threatening disease with high prevalence (2.4%), that is affecting about 68.56 million adult people (40+) in the world.

## Supplementary Information

Supplementary Information.
