# Supplementary material for: Prevalence of primary open angle glaucoma in the last 20 years: a meta-analysis and systematic review
Source: Sci Rep. 2021 Jul 2;11:13762. doi: 10.1038/s41598-021-92971-w (PMC8253788; doi:10.1038/s41598-021-92971-w)
Supplement: Supplementary file 1 — Supplementary Information. [file 41598_2021_92971_MOESM1_ESM.pdf]

Supplementary Materials

Title: Prevalence of Primary Open Angle Glaucoma in the Last 20 Years: A Meta-Analysis and Systematic Review

Authors: Nan Zhang[1,2 *], Jiaxing Wang[2 *], Ying Li[2] and Bing Jiang[1,3]

Affiliations: [1]Department of Ophthalmology, the Second Xiangya Hospital, Central South University, Changsha, Hunan, China
[2]Department of Ophthalmology, School of Medicine, Emory University, Atlanta, GA, United States
[3]Hunan Clinical Research Center of Ophthalmic Disease, Changsha, China
*: These authors contributed equally to this paper.

Corresponding Author: Bing Jiang, Department of Ophthalmology, the Second Xiangya Hospital, Central South University, Changsha, Hunan, China, 410011. E-mail: drjiangb@csu.edu.cn. Telephone number: 86 13875868866.

The study was conducted following the Preferred Reporting Items for guidelines of Systematic Reviews and Meta-analysis (PRISMA) guidelines[1, 2].

Table S1. Database specific search strategy.

Database	Search strategy
**PubMed**	1. ("glaucoma, open angle" [Mesh]) AND (("prevalence" [Mesh]) OR ("population" [Mesh]) OR ("epidemiology" [Mesh]) 2. (("primary open angle glaucoma" [Title/Abstract]) OR ("primary open-angle glaucoma" [Title/Abstract]) OR ("POAG" [Title/Abstract])) AND (("prevalence" [Title/Abstract]) OR ("Survey" [Title/Abstract]) OR ("population" [Title/Abstract]) OR ("epidemiology" [Title/Abstract])) 3. "2000"[Date - Publication]: "2020"[Date - Publication] (1 OR 2) AND 3
**Embase**	('primary open angle glaucoma': ab,ti OR 'primary open-angle glaucoma':ab,ti OR poag: ab,ti) AND (prevalence: ab,ti OR survey: ab,ti OR population: ab,ti OR epidemiology: ab,ti) AND [2000-2020]/py
**Web of Science**	1. TI= (((primary open angle glaucoma) OR (primary open-angle glaucoma) OR (POAG)) AND ((prevalence) OR (survey) OR (population) OR (epidemiology))) Timespan: 2000-2020. Databases: WOS, BCI, KJD, MEDLINE, RSCI, SCIELO. Search language=Auto 2. AB= (((primary open angle glaucoma) OR (primary open-angle glaucoma) OR (POAG)) AND ((prevalence) OR (survey) OR (population) OR (epidemiology))) Timespan: 2000-2020. Databases: WOS, BCI, KJD, MEDLINE, RSCI, SCIELO. Search language=Auto 1 OR 2

Table S2. Guidelines for assessing risk of bias – based on study participation and outcome measurement domains of the QUIPS tool [3].

**Potential bias**	**Items to be considered for assessment of potential bias**	**Items for risk ratings**
**Study participation**<br>Does the study sample represent the population of interest?	1. Description of the baseline study sample. Is this study population based and randomized sampling?<br>2. Description of the source population or population of interest<br>3. Adequate description of the source population of interest, the sampling frame and place of recruitment<br>4. Is there adequate participation in the study?<br>5. Response rate<br>6. Participants selection biased by potential risk factors for POAG, such as age, gender and ethnicity.	**High risk of bias:**<br>The relationship between the PF and outcome is very likely to be different for<br>participants and eligible nonparticipants<br>Age inclusion of only 60+<br>**Moderate risk of bias**<br>The relationship between the PF and outcome may be different for participants<br>and eligible nonparticipants<br>Only a certain minority group was included<br>Age inclusion of only 50+<br>**Low risk of bias**<br>The relationship between the PF and outcome is unlikely to be different for participants and eligible nonparticipants<br>Age inclusion of 40+
**Outcome measurement**<br>Is the outcome of interest measured in a similar<br>way for all participants?	1. Whether the ISGEO2002 diagnosis criteria were used for definition of POAG<br>2. Whether a clear definition of POAG is provided if ISGEO2002 criteria is not used in the study<br>3. Whether the method of POAG definition used is adequately valid and reliable<br>4. Whether the method is the same for all study participants<br>5. Is there possible involvement of other types of open angle glaucoma (OAG) in the reported prevalence?	**High risk of bias:**<br>The measurement of the PF is very likely<br>to be different for different levels of the outcome of interest<br>**Moderate risk of bias**<br>The measurement of the PF may be different for different levels of the outcome of interest<br>**Low risk of bias**<br>The measurement of the PF is unlikely<br>to be different for different levels of the outcome of interest

Table S3. Risk of selection and outcome measurement bias and justification for rating using the QUIPS tool.

Study	*Risk of study participation bias*	*Risk of outcome measurement bias*
Bonomi, 2000, Italy [21]	**Moderate**<br>Randomized, population-based sampling for subjects with age of 40+. Response rate was relatively low at 74% and therefore considered moderate risk for selection bias.	**Moderate**<br>Prevalence of POAG was reported. Diagnosis criteria of ISGEO2002 was not used and therefore considered of moderate risk for outcome measurement bias. The diagnosis of POAG was made on the basis of the concomitant presence of at least two of the following criteria: intraocular pressure ≥ 22 mmHg, glaucomatous optic disc abnormalities, glaucomatous visual field defects.
Buhrmann, 2000, Tanzania [22]	**Low**<br>Randomized, population-based sampling for subjects with age of 40+. Response rate was 90% and therefore considered low risk for selection bias.	**Moderate**<br>Prevalence of POAG was reported. Diagnosis criteria of ISGEO2002 was not used and therefore considered of moderate risk for outcome measurement bias. The definition of primary OAG for this survey depended on the grading of the optic disc by the ophthalmologist, the visual field finding, the absence of an occludable angle, and the absence of a secondary cause for glaucoma.
Dandona, 2000, India [23]	**Moderate**<br>Stratified random sampling with equal probability of selection. Response rate was 90%. The included participants aged from 16 years old and over. The overall prevalence of POAG were likely to be underestimated for the population of 40+ years of age and therefore considered moderate risk for selection bias.	**Moderate**<br>Prevalence of POAG was reported. Diagnosis criteria of ISGEO2002 was not used and therefore considered of moderate risk for outcome measurement bias.<br>Definite POAG was defined as the presence of glaucomatous optic disc damage along with visual field loss consistent with the disc findings, in the presence of an open-angle.
Foster, 2000, Singapore [24]	**Moderate**<br>Disproportionate, stratified, clustered, random-sampling for subjects with age of 40+. Response rate was relatively low at 71.8% and therefore considered moderate risk for selection bias.	**Moderate**<br>Prevalence of POAG was reported. Diagnosis criteria of ISGEO2002 was not used and therefore considered of moderate risk for outcome measurement bias.<br>Full ocular examination including visual acuity, visual field, near-refractive correction, screening-mode frequency doubling technology test, anterior segment, IOP, Gonioscopy and fundus were performed. Very detailed diagnostic criteria were provided in the study.

Quigley, 2001, USA [25]	**Moderate**<br>Randomized, population-based sampling for subjects with age of 40+. Response rate was relatively low at 74% and therefore considered moderate risk for selection bias.	**Moderate**<br>Prevalence of POAG was reported. Diagnosis criteria of ISGEO2002 was not used and therefore considered of moderate risk for outcome measurement bias.<br>The criteria for OAG were those to be published soon by an international group that considered a definitional structure at the 1998 meeting of the International Society for Geographic and Epidemiologic Ophthalmology, June 14, 1998, Leeuwenhorst, the Netherlands.
Weih, 2001, Australia [26]	**Low**<br>Cluster-stratified random sampling for subjects with age of 40+. Response rate was 86% and therefore considered low risk for selection bias.	**High**<br>ISGEO2002 criteria was not used for diagnose. Diagnosis of glaucoma was by consensus of a group panel of six ophthalmologists, two of whom were glaucoma subspecialists.26 Glaucoma suspects were defined as participants who had IOP of more than 21 mmHg, a glaucomatous visual field defect, C/D ratio more than 0.7 in either eye, C/D ratio asymmetry more than 0.3, or who reported a history of glaucoma (either diagnosis or treatment).<br>The prevalence of OAG instead of POAG was reported. The involvement of other types of OAG led to an overestimation of the prevalence of POAG, and therefore considered to be at high risk for outcome measurement.
Rotchford, 2002, South Africa [27]	**Low**<br>Two-stage, non-stratified, cluster-based, random-sampling for subjects with age of 40+. Response rate was 90% and therefore considered low risk for selection bias.	**Low**<br>Prevalence of POAG was reported. ISGEO2002 criteria was used for diagnose. Therefore, considered as low risk for outcome measurement bias
Bourne, 2003, Thailand [28]	Moderate<br>Randomized, population-based sampling for subjects with age of 50+. The sampling process requires the individuals in a household had to own the home<br>and individuals selected had to have no intention of moving from the area within 3 years. Therefore, this study was considered moderate risk for selection bias. Response rate was high at 88.5%.	**Low**<br>Prevalence of POAG was reported. ISGEO2002 criteria was used for diagnose. Therefore, considered as low risk for outcome measurement bias

Jonasson, 2003, Iceland [29]	**Moderate**<br>Randomized, population-based sampling for subjects with age of 50+ and therefore was considered moderate risk for selection bias. Response rate was high at 91%.	**High**<br>ISGEO2002 criteria was used for diagnose. The prevalence of OAG instead of POAG was reported. The involvement of other types of OAG led to an overestimation of the prevalence of POAG, and therefore considered to be at high risk for outcome measurement.
Ramakrishnan, 2003, India [30]	**Low**<br>Two-stage cluster sampling for subjects with age of 40+. Response rate was 96.5% and therefore considered low risk for selection bias.	**Moderate**<br>Prevalence of POAG was reported. Diagnosis criteria of ISGEO2002 was not used and therefore considered of moderate risk for outcome measurement bias. Definite POAG was defined as angles open on gonioscopy and glaucomatous optic disc changes with matching visual field defects, whereas ocular hypertension was defined as intraocular pressure (IOP) greater than 21 mmHg without glaucomatous optic disc damage and visual field defects in the presence of an open angle.
Rotchford, 2003, South Africa [31]	**Moderate**<br>Two-stage non-stratified, cluster-based random sampling for subjects with age of 40+. Response rate was relatively low at 74% and therefore considered moderate risk for selection bias.	**Low**<br>Prevalence of POAG was reported. ISGEO2002 criteria was used for diagnose. Therefore, considered as low risk for outcome measurement bias
Anton, 2004, Spanish [32]	**Moderate**<br>Randomized, population-based sampling for subjects with age of 40+. Response rate was not reported and therefore considered moderate risk for selection bias.	**Moderate**<br>Prevalence of POAG was reported. Diagnosis criteria of ISGEO2002 was not used and therefore considered of moderate risk for outcome measurement bias. Definite POAG was defined as IOP >21 mm Hg; Glaucomatous Optic Disk; Glaucomatous Visual Field and open angle.
Iwase, 2004, Japan [33]	**Low**<br>Simple random sampling without stratification for subjects with age of 40+. Response rate was 78.1% and therefore considered low risk for selection bias.	**Low**<br>Prevalence of POAG was reported. ISGEO2002 criteria was used for diagnose. Therefore, considered as low risk for outcome measurement bias
Ntim-Amponsah, 2004, Ghnana [34]	**Moderate**<br>Randomized, population-based sampling for subjects with age of 40+. Response rate was not reported and therefore considered moderate risk for selection bias.	**Moderate**<br>Prevalence of POAG was reported. Diagnosis criteria of ISGEO2002 was not used and therefore considered of moderate risk for outcome measurement bias. The diagnosis of glaucoma was based on glaucomatous optic nerve damage,

		including abnormal visual fields and/or optic disc cupping with or without elevated IOP (by Perkin's applanation tonometer)."
Rahman, 2004, Bangladesh [35]	**Moderate**<br>Multistage, stratified, clustered sampling for subjects with age of 35+. Response rate was low of 66% and therefore considered moderate risk for selection bias.	**Low**<br>Prevalence of POAG was reported. ISGEO2002 criteria was used for diagnose. Therefore, considered as low risk for outcome measurement bias
Varma, 2004, USA [36]	**Low**<br>Randomized, population-based sampling for subjects with age of 40+. Response rate was 82% and therefore considered low risk for selection bias.	**High**<br>ISGEO2002 criteria was not used for diagnose. Open-angle glaucoma was defined as the presence of an open angle and various criteria that included a glaucomatous VF abnormality and/or evidence of glaucomatous optic disc damage in at least one eye.<br>The prevalence of OAG instead of POAG was reported. The involvement of other types of OAG led to an overestimation of the prevalence of POAG, and therefore considered to be at high risk for outcome measurement.
Nizankowska, 2005, Poland [37]	**Low**<br>Proportional simple random sampling selection with stratification for subjects with age of 40+. Response rate was 81% and therefore considered low risk for selection bias.	**Moderate**<br>Prevalence of POAG was reported. Diagnosis criteria of ISGEO2002 was not used and therefore considered of moderate risk for outcome measurement bias. Glaucoma was diagnosed by the presence of any two of the following: characteristic morphological changes in the optic disc, glaucomatous visual field abnormalities, and intraocular pressure greater than 21 mmHg.
Raychaudhuri, 2005, India [38]	**Moderate**<br>Randomized, population-based sampling for subjects with age of 50+ and therefore was considered moderate risk for selection bias. Response rate was high at 83.1%.	**Low**<br>Prevalence of POAG was reported. ISGEO2002 criteria was used for diagnose. Therefore, considered as low risk for outcome measurement bias
Vijaya, 2005, India [39]	**Moderate**<br>Nonrandom sample was enumerated from a defined population and therefore considered low risk for selection bias. However, this sampling process has included almost all of the people aged over 40 years old that live in a particular rural area. This leads to limited bias for	**Low**<br>Prevalence of POAG was reported. ISGEO2002 criteria was used for diagnose. Therefore, considered as low risk for outcome measurement bias

	the prevalence of POAG and therefore included in this review. Their response rate was 81.75%.	
Yamamoto, 2005, Japan [40]	**Low**<br>Randomized, population-based sampling for subjects with age of 40+. Response rate was 78.1% and therefore considered low risk for selection bias.	**Low**<br>Prevalence of POAG was reported. ISGEO2002 criteria was used for diagnose. Therefore, considered as low risk for outcome measurement bias
Friedman, 2006, England [41]	**High**<br>Randomized, population-based sampling for subjects with age of 73+. The selection of aged people has led to an overestimation of prevalence for their population and therefore considered to be at high risk for selection bias.<br>Response rate was 98.6%.	**High**<br>1.The prevalence of OAG instead of POAG was reported. The involvement of other types of OAG led to an overestimation of the prevalence of POAG.<br>2.ISGEO2002 criteria was not used for diagnose. Glaucoma was classified as OAG if the pigmented trabecular meshwork was visible for greater than 90° without compression and there were no peripheral anterior synechiae or if peripheral anterior synechiae were present but prior surgery had been performed.<br>In this study, visual field testing was mot performed on the subjects because the glaucoma grading scheme required an enlarged VCDR for a person to be classified as having glaucoma. Incomplete field testing would bias the results toward an underestimation of the prevalence of glaucoma.<br>Overall, this study was considered to be at high risk for outcome measurement.
He, 2006, China [42]	**Moderate**<br>Random clustering sampling for subjects with age of 50+ and therefore was considered moderate risk for selection bias. Response rate was high at 75.3%.	**Low**<br>Prevalence of POAG was reported. ISGEO2002 criteria was used for diagnose. Therefore, considered as low risk for outcome measurement bias
Casson, 2007, Myanmar [43]	**Low**<br>Randomized, population-based sampling for subjects with age of 40+. Response rate was 80.5% and therefore considered low risk for selection bias.	**Low**<br>Prevalence of POAG was reported. ISGEO2002 criteria was used for diagnose. Therefore, considered as low risk for outcome measurement bias
Sakata, 2007, Brazil [44]	**Low**<br>Randomized, population-based sampling for subjects with age of 40+. Response rate was 76.5% and	**Low**<br>Prevalence of POAG was reported. ISGEO2002 criteria was used for diagnose. Therefore, considered as low risk for outcome measurement bias

	therefore considered low risk for selection bias.	
Topouzis, 2007, Greece [45]	**Moderate**<br>Randomized, population-based sampling for subjects with age of 40+. Response rate was low at 71% and therefore considered moderate risk for selection bias.	**Moderate**<br>Prevalence of POAG was reported. Diagnosis criteria of ISGEO2002 was not used and therefore considered of moderate risk for outcome measurement bias. Definition of glaucoma was based on the presence of both glaucomatous optic disk and confirmed glaucomatous visual field defect. Subjects also were classified as having glaucoma when the clinical judgment was strongly in favor of the presence of glaucoma even though the strict criteria were not fulfilled.
Shen, 2008, Singapore [46]	**Low**<br>Age-stratified, random sampling for subjects with age of 40+. Response rate was 78.7% and therefore considered low risk for selection bias.	**Low**<br>Prevalence of POAG was reported. ISGEO2002 criteria was used for diagnose. Therefore, considered as low risk for outcome measurement bias
Vijaya, 2008, India [47]	**Low**<br>Multistage random cluster sampling for subjects with age of 40+. Response rate was 80.2% and therefore considered low risk for selection bias.	**Low**<br>Prevalence of POAG was reported. ISGEO2002 criteria was used for diagnose. Therefore, considered as low risk for outcome measurement bias
Garudadri, 2010, India [48]	**Low**<br>Stratified, random, cluster, and systematic sampling for subjects with age of 40+. Response rate was 88% and therefore considered low risk for selection bias.	**Low**<br>Prevalence of POAG was reported. ISGEO2002 criteria was used for diagnose. Therefore, considered as low risk for outcome measurement bias
kuzin, 2010, USA [49]	**Low**<br>Noninstitutionalized, random, population-based sampling for subjects with age of 40+. Response rate was 82% and therefore considered low risk for selection bias.	**High**<br>ISGEO2002 criteria was not used for diagnose. Open-angle glaucoma was defined by the presence of an open angle and at least one of the following criteria: (1) congruent, characteristic, or compatible glaucomatous visual field abnormality or (2) evidence of characteristic or compatible glaucomatous optic disc damage in at least 1 eye, as determined by 2 independent glaucoma specialist graders who reviewed all cases.<br>The prevalence of OAG instead of POAG was reported. The involvement of other types of OAG led to an overestimation of the prevalence of POAG, and therefore

		considered to be at high risk for outcome measurement.
Sia, 2010, Sri Lanka [50]	**Low**<br>Randomized, population-based sampling for subjects with age of 40+. Response rate was 80% and therefore considered low risk for selection bias.	**Low**<br>Prevalence of POAG was reported. ISGEO2002 criteria was used for diagnose. Therefore, considered as low risk for outcome measurement bias
Wang, 2010, China [51]	**Low**<br>Randomized, population-based sampling for subjects with age of 40+. Response rate was 83.4% and therefore considered low risk for selection bias.	**High**<br>ISGEO2002 criteria was used for diagnose. The prevalence of OAG instead of POAG was reported. The involvement of other types of OAG led to an overestimation of the prevalence of POAG, and therefore considered to be at high risk for outcome measurement.
Al-Mansouri, 2011, Qatar [52]	**Low**<br>Random selected clusters for subjects with age of 40+. Response rate was 97.3% and therefore considered low risk for selection bias.	**Moderate**<br>Prevalence of POAG was reported. Diagnosis criteria of ISGEO2002 was not used and therefore considered of moderate risk for outcome measurement bias. Glaucoma was defined as either eye had (1) evidence of glaucomatous cup or surrounding retina showed signs of glaucoma and (2) intraocular pressure of ≥22 mmHg measured with applanation tonometry
Kim, 2011, south Korea [53]	**Low**<br>Randomized, population-based sampling for subjects with age of 40+. Response rate was 79.5% and therefore considered low risk for selection bias.	**Low**<br>Prevalence of POAG was reported. ISGEO2002 criteria was used for diagnose. Therefore, considered as low risk for outcome measurement bias
Liang, 2011, China [54]	**Low**<br>Randomized, population-based sampling for subjects with age of 40+. Response rate was 98.3% and therefore considered low risk for selection bias.	**Low**<br>Prevalence of POAG was reported. ISGEO2002 criteria was used for diagnose. Therefore, considered as low risk for outcome measurement bias
Song, 2011, China [55]	**Low**<br>Randomized, population-based sampling for subjects with age of 40+. Response rate was 87.4% and therefore considered low risk for selection bias.	**Low**<br>Prevalence of POAG was reported. ISGEO2002 criteria was used for diagnose. Therefore, considered as low risk for outcome measurement bias
Thapa, 2012, Nepal [56]	**Low**<br>Randomized, cluster sampling for subjects with age of 40+. Response rate was 83.4% and therefore	**Low**<br>Prevalence of POAG was reported. ISGEO2002 criteria was used for diagnose.

	considered low risk for selection bias.	Therefore, considered as low risk for outcome measurement bias
Zhong, 2012, China [57]	**Moderate** Randomized, population-based sampling for subjects with age of 50+ and therefore was considered moderate risk for selection bias. Response rate was high at 77.8%.	**Low** Prevalence of POAG was reported. ISGEO2002 criteria was used for diagnose. Therefore, considered as low risk for outcome measurement bias
Ashaye, 2013, Nigeria [58]	**Low** Randomized, population-based sampling for subjects with age of 40+. Response rate was 90% and therefore considered low risk for selection bias.	**Low** Prevalence of POAG was reported. ISGEO2002 criteria was used for diagnose. Therefore, considered as low risk for outcome measurement bias
Budenz, 2013, Ghana [59]	**Low** Randomly selected clusters sampling for subjects with age of 40+. Response rate was 82.3% and therefore considered low risk for selection bias.	**Low** Prevalence of POAG was reported. ISGEO2002 criteria was used for diagnose. Therefore, considered as low risk for outcome measurement bias
Narayanaswamy, 2013, Singapore [60]	**Low** Age-stratified random sampling for subjects with age of 40+. Response rate was 75.6% and therefore considered low risk for selection bias.	**Low** Prevalence of POAG was reported. ISGEO2002 criteria was used for diagnose. Therefore, considered as low risk for outcome measurement bias
Pakravan, 2013, Iran [61]	**Low** Randomized, cluster sampling for subjects with age of 40+. Response rate was 90.4% and therefore considered low risk for selection bias.	**Low** Prevalence of POAG was reported. ISGEO2002 criteria was used for diagnose. Therefore, considered as low risk for outcome measurement bias
Yamamoto, 2014, Japan [62]	**Low** Randomized, population-based sampling for subjects with age of 40+. Response rate was 81.1% and therefore considered low risk for selection bias.	**Low** Prevalence of POAG was reported. ISGEO2002 criteria was used for diagnose. Therefore, considered as low risk for outcome measurement bias
Baskaran, 2015, Singapore [63]	**Moderate** Age-stratified random sampling for subjects with age of 40+. Response rate was 72.8% and therefore considered moderate risk for selection bias.	**Low** Prevalence of POAG was reported. ISGEO2002 criteria was used for diagnose. Therefore, considered as low risk for outcome measurement bias
He, 2015, China [64]	**Moderate** Randomized, population-based sampling for subjects with age of 50+ and therefore was considered	**Low** Prevalence of POAG was reported. ISGEO2002 criteria was used for diagnose. Therefore, considered as low risk for outcome measurement bias

	moderate risk for selection bias. Response rate was 80.36%.	
Kyari, 2015, Nigeria [65]	**Low** Multistage stratified cluster random sampling for subjects with age of 40+. Response rate was 90.4% and therefore considered low risk for selection bias.	**Low** Prevalence of POAG was reported. ISGEO2002 criteria was used for diagnose. Therefore considered as low risk for outcome measurement bias
Kim, 2016, South Korea [66]	**Moderate** Complex, stratified, multistage, probability-cluster sampling for subjects with age of 40+ and therefore considered low risk for selection bias. Response rate was 76.7%.	**Low** Prevalence of POAG was reported. ISGEO2002 criteria was used for diagnose. Therefore, considered as low risk for outcome measurement bias
Paul, 2016, India [67]	**Low** Multistage random cluster sampling for subjects with age of 40+. Response rate was 98% and therefore considered low risk for selection bias.	**Low** Prevalence of POAG was reported. ISGEO2002 criteria was used for diagnose. Therefore, considered as low risk for outcome measurement bias
Chassid, 2018, Israel [68]	**Low** Randomized, population-based sampling for subjects with age of 40+. Response rate was 82% and therefore considered low risk for selection bias.	**Moderate** Prevalence of POAG was reported. Diagnosis criteria of ISGEO2002 was not used and therefore considered of moderate risk for outcome measurement bias. Individuals were classified with POAG if they met any of the following diagnostic criteria: cup-to-disk ratio (CDR) >0.5 with asymmetry between the eyes and damage to the field of vision; intraocular pressure (IOP) >23 mm Hg; damage to the field of vision without CDR asymmetry, but with IOP >23 mm Hg; CDR asymmetry >0.5 with damage to the field of vision, independent of IOP; CDR asymmetry >0.2 and damage to the field of vision, with or without increased IOP; damage to the field of vision as defined by the following characteristics of glaucomatous field defects: asymmetry across the horizontal midline (in early/moderate cases), location in the mid-periphery (in early/moderate cases), clustering in neighboring test points, and reproducibility on at least two occasions.
Addepalli, 2019, India [69]	**Low** Randomized, population-based sampling for subjects with age of 40+. Response rate was 86% and	**Low** Prevalence of POAG was reported. ISGEO2002 criteria was used for diagnose.

	therefore considered low risk for selection bias.	Therefore, considered as low risk for outcome measurement bias
McCann, 2020, Northern Ireland [70]	**Moderate**<br>Randomized, population-based sampling for subjects with age of 40+. Response rate was not reported and therefore considered moderate risk for selection bias.	**Low**<br>Prevalence of POAG was reported. ISGEO2002 criteria was used for diagnose. Therefore, considered as low risk for outcome measurement bias

1. Liberati, A., et al., *The PRISMA statement for reporting systematic reviews and meta-analyses of studies that evaluate health care interventions: explanation and elaboration.* PLoS Med, 2009. **6**(7): p. e1000100.
2. Moher, D., et al., *Preferred reporting items for systematic reviews and meta-analyses: the PRISMA statement.* PLoS Med, 2009. **6**(7): p. e1000097.
3. Hayden, J.A., et al., *Assessing bias in studies of prognostic factors.* Ann Intern Med, 2013. **158**(4): p. 280-6.

Table S4. Characteristics of included studies.

Supp. Table 4. Characteristics of included studies.

Reference#	PMID	Author	Year of Publication	Continent	Ethnity	Country	Hibitation area	Age range	No. of Participants	No. of cases	Crude prevalence (95%CI)	Response rate
21	10811096	Bonomi	2000	Europe	Europe	Italy	Mixed	40+	4297	84	1.90 (1.50; 2.40)	74%
22	10634599	Buhrmann	2000	Africa	Africa	Tanzania	Rural	40+	3268	100	3.08 (2.50; 3.80)	90%
23	10964833	Dandona	2000	Asia	Asia	India	Urban	16+	2522	27	1.07 (0.67; 1.47)	85%
24	10922206	Foster	2000	Asia	Asia	Singapore	Unknown	40+	1232	22	1.79 (1.05; 2.53)	72%
25	11735794	Quigley	2001	N. America	Hispanic	USA	Unknown	40+	4774	94	1.97 (1.57; 2.36)	72%
26**	11713063	Weih	2001	Oceania	Australia	Australia	Urban	40+	3264	56	1.72 (1.27; 2.16)	86%
							rural	40+	1369	29	2.12 (1.36; 2.88)	
27	11934321	Rotchford	2002	Africa	Africa	South Africa	Rural	40+	1005	28	2.70 (1.70; 4.00)	90%
28	12928267	Bourne	2003	Aisa	Asia	Thailand	Urban	50+	790	16	2.03 (1.04; 3.01)	89%
29**	12928689	Jonasson	2003	Europe	Europe	Iceland	Unknown	50+	1045	42	4.02 (2.83; 5.21)	91%
30	12917161	Ramakrishna n	2003	Aisa	Asian	India	Rural	40+	5150	64	1.20 (0.90; 1.50)	97%
31	12578784	Rotchford	2003	Africa	African	South Africa	Urban	40+	839	31	2.90 (1.90; 4.30)	75%
32	15354074	Anton	2004	Europe	Europe	Spanish	Unknown	40+	569	12	2.11 (0.93; 3.29)	Not reported
33	15350316	Iwase	2004	Aisa	Asia	Japan	Unknown	40+	3021	119	3.90 (3.30; 4.60)	78%
34	15131680	Ntim-Amponsah	2004	Africa	Africa	Ghnana	Unknown	30+	1785	148	8.29 (7.01; 9.57)	Not reported
35	15548796	Rahman	2004	Aisa	Asia	Bangladesh	Unknown	35+	2347	29	1.24 (0.79; 1.68)	66%
36**	15288969	Varma	2004	N.America	Latino	USA	Unknown	40+	6142	291	4.74 (4.22; 5.30)	82%
37	16283988	Nizankowska	2005	Europe	Europe	Poland	Unknown	40+	4853	49	1.01(0.73; 1.29)	81%
38	16299129	Raychaudhur i	2005	Aisa	Asia	India	Rural	50+	1324	38	2.87 (1.97; 3.77)	83%
39	16303934	Vijaya	2005	Aisa	Asia	India	Rural	40+	3924	64	1.62 (1.42; 1.82)	82%
40	16111758	Yamamoto	2005	Aisa	Asia	Japan	Unknown	40+	3021	118	3.91 (3.22; 4.60)	78%
41**	17102012	Friedman	2006	Europe	Mixed	England	Unknown	70+	1214	136	11.20 (9.43; 12.98)	96%
42	16799014	He	2006	Aisa	Asia	China	urban	50+	1405	29	2.06 (1.32; 2.81)	75%
43	17510475	Casson	2007	Asia	Asia	Myanmar	Rural	40+	2076	42	2.00 (0.90; 3.10)	81%
44	17962447	Sakata	2007	S.America	Mixed	Brazil	Unknown	40+	1636	40	2.44 (1.70; 3.19)	77%
45	17893012	Topouzis	2007	Europe	Europe	Greece	Urban	40+	2554	69	2.70 (2.07; 3.33)	71%

Table S4. Characteristics of included studies. (continued)

Supp. Table 4. (continued)

Reference#	PMID	Author	Year of Publication	Continent	Ethnity	Country	Hibitation area	Age range	No. of Participants	No. of cases	Crude prevalence (95%CI)	Response rate
46	18441307	Shen	2008	Aisa	Asia	Singapore	Urban	40+	3280	104	3.20 (2.60; 3.80)	79%
47	17664010	Vijaya	2008	Aisa	Asia	India	Urban	40+	3850	135	3.51 (2.93; 4.09)	80%
48	20188420	Garudadri	2010	Asia	Asia	India	Urban	40+	934	37	4.00 (2.74; 5.25)	88%
							Rural		2790	45	1.60 (1.13; 2.06)	85%
49**	20570359	kuzin	2010	N.America	latino	USA	Unknown	40+	5927	252	4.25 (3.74; 4.77)	82%
50	20642343	Sia	2010	Aisa	Asia	Sri Lanka	Rural	40+	1375	32	2.30 (1.50; 3.20)	80%
51**	20970107	Wang	2010	Aisa	Asia	China	Mixed	40+	4439	111	2.60 (2.12; 3.08)	83%
52	21731325	Al- Mansouri	2011	Aisa	Asia	Qatar	Unknown	40+	3149	44	1.40 (0.99; 1.81)	97%
53	21269703	kim	2011	Asia	Asia	south korea	Rural	40+	1532	55	3.60 (2.60; 4.50)	80%
54	21896871	Liang	2011	Aisa	Asia	China	Rural	30+	6716	67	1.00 (0.76; 1.24)	98%
55	21684607	Song	2011	Aisa	Asia	China	Rural	40+	5158	73	1.42 (1.09-1.74)	87%
56	22305097	Thapa	2012	Aisa	Asia	Nepal	Unknown	40+	3991	51	1.24 (1.14; 1.34)	83%
57	22511635	Zhong	2012	Aisa	Asia	China	Rural	50+	2133	22	1.00 (0.60; 1.60)	78%
58	24135752	Ashaye	2013	Africa	Africa	Nigeria	Unknown	40+	811	50	6.20 (4.50; 7.80)	90%
59	23538512	Budenz	2013	Africa	Africa	Ghana	Urban	40+	5603	342	6.80 (6.20; 7.40)	82%
60	23745009	Narayanasw amy	2013	Asia	Asia	Singapore	Urban	40+	3400	46	1.35 (0.96; 1.74)	76%
61	23664464	Pakravan	2013	Asia	Asia	Iran	Mixed	40+	2320	64	3.20 (2.30; 4.20)	90%
62	24746386	Yamamoto	2014	Aisa	Asia	Japan	Rural	40+	3762	151	4.01 (3.39; 4.64)	81%
63	25974263	Baskaran	2015	Aisa	Aisa	Singapore	Urban	40+	3353	57	1.70 (1.26; 2.14)	73%
64	26462564	He	2015	Aisa	Asia	China	Urban	50+	2528	72	2.85 (2.20; 3.50)	80%
65	26653326	Kyari	2015	Africa	Africa	Nigeria	Mixed	40+	13591	208	1.53 (1.32; 1.74)	90%
66	26746594	kim	2016	Aisa	Asia	South korea	Mixed	40+	13831	710	5.13 (4.77; 5.50)	77%
67	27688279	Paul	2016	Aisa	Asia	India	Urban	40+	7128	150	2.10 (1.77; 2.44)	98%
							Rural	40+	6964	101	1.45 (1.17; 1.73)	94%
68	29376006	Chassid	2018	Aisa	Asia	Israel	Unknown	30+	15122	454	3.00 (2.73; 3.27)	82%
69	30489169	Addepalli	2019	Aisa	Asia	India	Unknown	40+	3833	41	1.07 (0.74; 0.39)	Not reported
70	32034006	McCann	2020	Europe	Europe	Northern Ireland	Mixed	50+	3221	48	1.49 (1.07; 1.91)	63%

POAG= primary open-angle glaucoma; S. America= South America; N. America= North America; **: Studies of open-angle glaucoma.

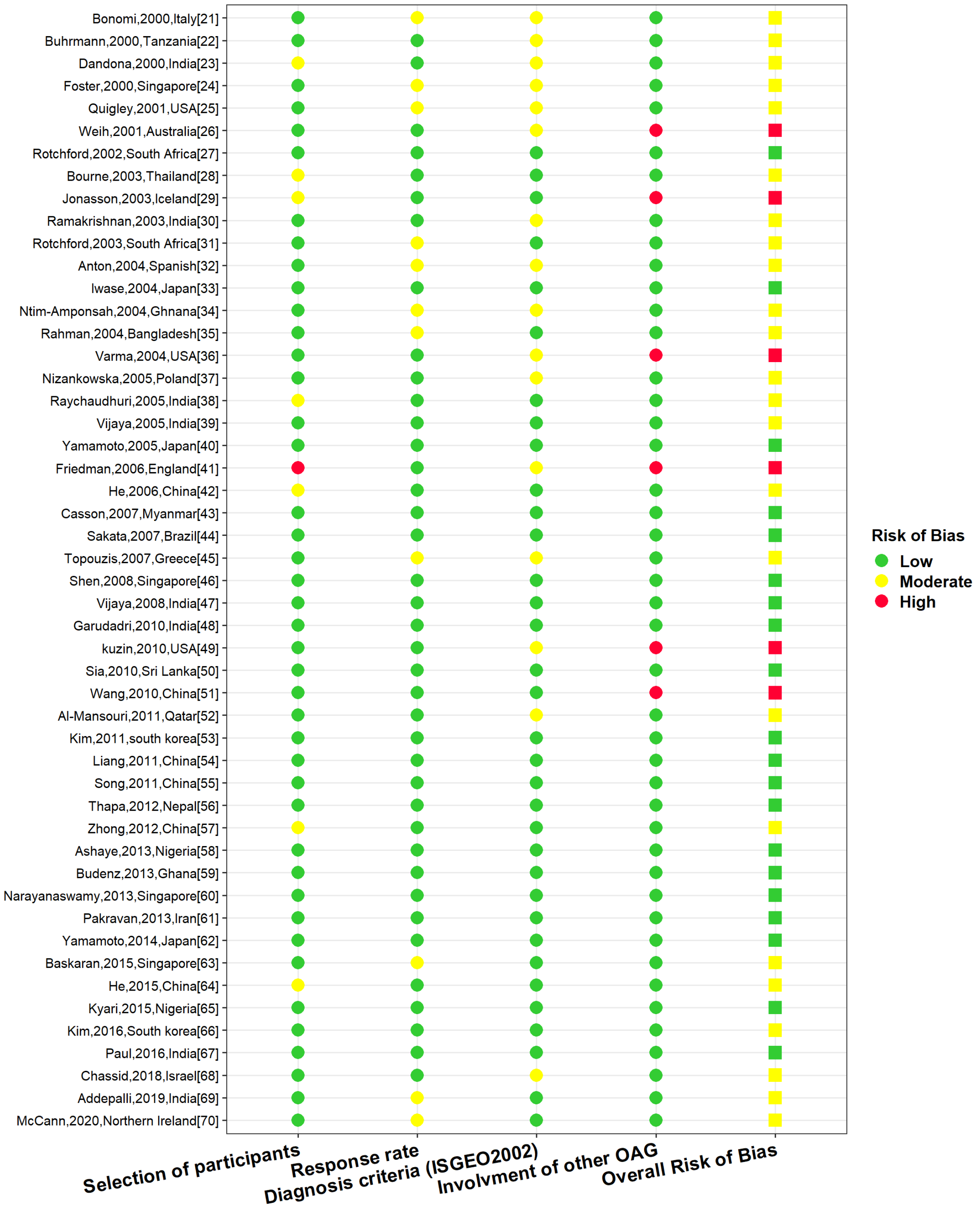

Figure S1. Traffic lights for risk of bias.

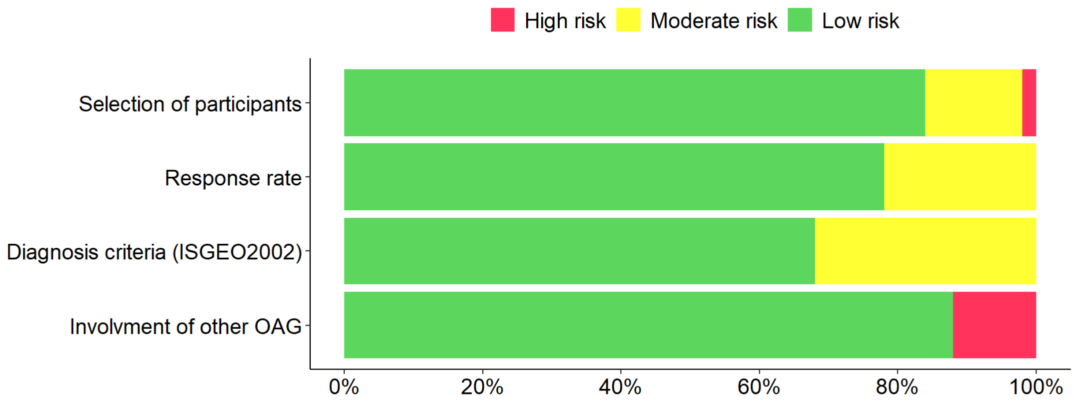

Figure S2. Summary of risk of bias.

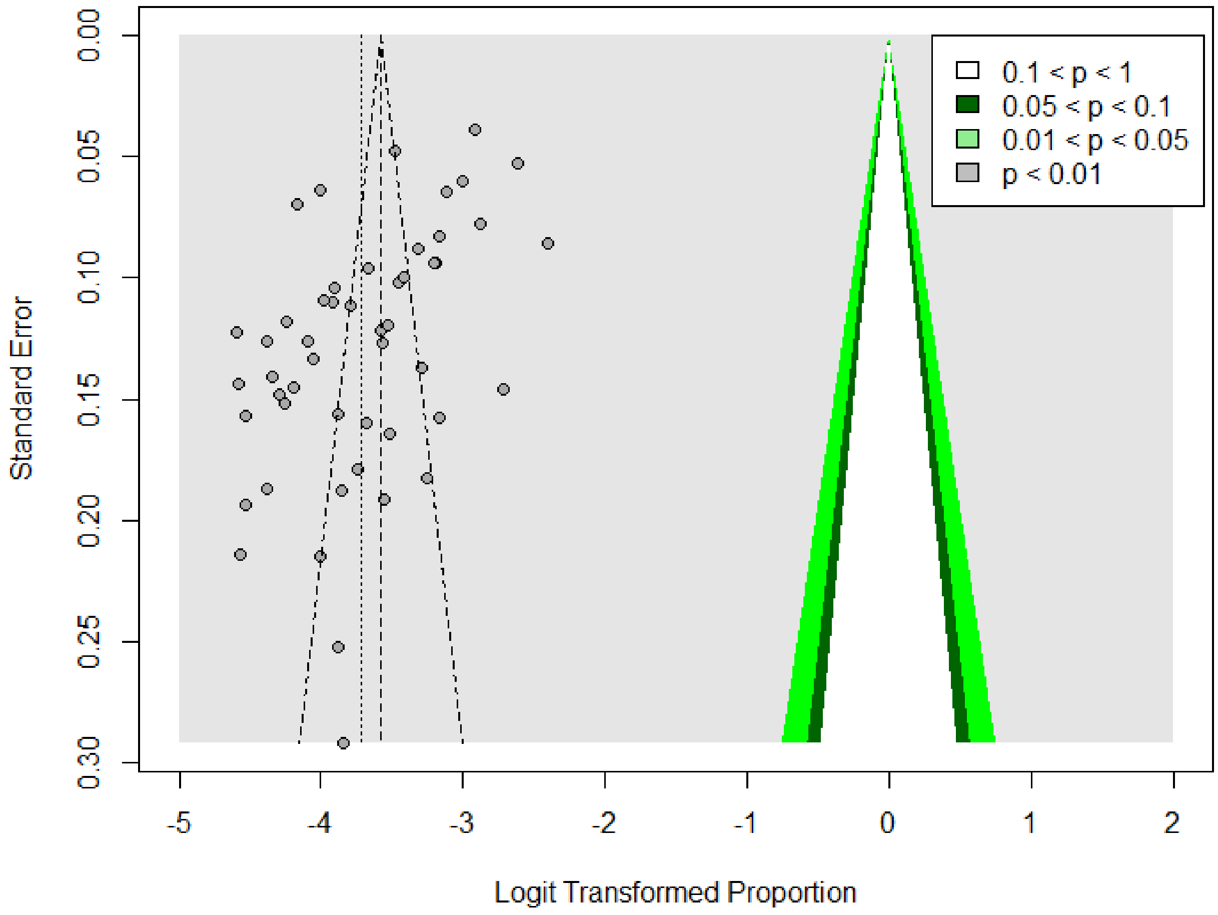

Figure S3. Funnel plots for all studies.

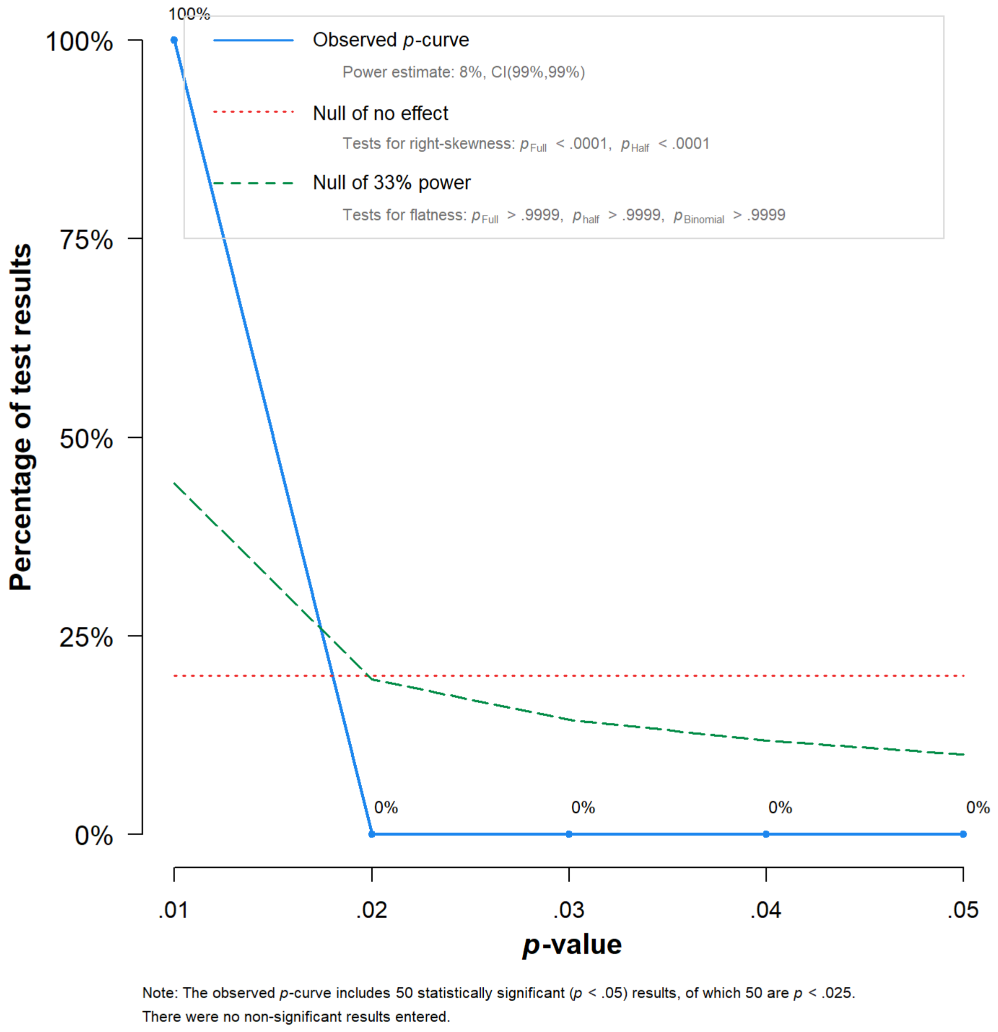

Figure S4. P-value analysis for all studies.

Study	Glaucoma	Total	Prevalence	95% CI
**Female**				
Bonomi, 2000, Italy [21]	46	2415	0.019	[0.014; 0.024]
Dandona, 2000, India [23]	14	1347	0.010	[0.005; 0.016]
Foster, 2000, Singapore [24]	7	675	0.010	[0.003; 0.018]
Quigley, 2001, USA [25]	61	2923	0.021	[0.016; 0.026]
Weih, 2001, Australia [26**]	45	2523	0.018	[0.013; 0.023]
Bourne, 2003, Thailand [28]	13	494	0.026	[0.012; 0.040]
Jonasson, 2003, Iceland [29**]	20	584	0.034	[0.019; 0.049]
Ramakrishnan , 2003, India [30]	28	2836	0.010	[0.006; 0.014]
Iwase, 2004, Japan [33]	62	1687	0.037	[0.028; 0.046]
Rahman, 2004, Bangladesh [35]	10	1127	0.009	[0.003; 0.014]
Varma, 2004, USA [36**]	152	3494	0.044	[0.037; 0.050]
Nizankowska, 2005, Poland [37]	28	2821	0.010	[0.006; 0.014]
Raychaudhuri, 2005, India [38]	16	695	0.023	[0.012; 0.034]
Vijaya, 2005, India [39]	34	2163	0.016	[0.010; 0.021]
Yamamoto, 2005, Japan [40]	63	1687	0.037	[0.028; 0.046]
He, 2006, China [42]	8	792	0.010	[0.003; 0.017]
Sakata, 2007, Brazil [44]	17	940	0.018	[0.010; 0.027]
Shen, 2008, Singapore [46]	45	1704	0.026	[0.019; 0.034]
Vijaya, 2008, India [47]	71	2140	0.033	[0.026; 0.041]
Garudadri, 2010, India [48]	44	1973	0.022	[0.016; 0.029]
kuzin, 2010, USA [49**]	126	3448	0.037	[0.030; 0.043]
Wang, 2010, China [51**]	48	2400	0.020	[0.014; 0.026]
Kim, 2011, South Korea [53]	27	887	0.030	[0.019; 0.042]
Liang, 2011, China [54]	41	3599	0.011	[0.008; 0.015]
Song, 2011, China [55]	40	2859	0.014	[0.010; 0.018]
Thapa, 2012, Nepal [56]	25	2172	0.012	[0.007; 0.016]
Ashaye, 2013, Nigeria [58]	19	422	0.045	[0.025; 0.065]
Budenz, 2013, Ghana [59]	166	3379	0.049	[0.042; 0.056]
Narayanaswamy, 2013, Singapore [60]	18	1694	0.011	[0.006; 0.016]
Baskaran, 2015, Singapore [63]	16	1691	0.009	[0.005; 0.014]
He, 2015, China [64]	35	1460	0.024	[0.016; 0.032]
kim, 2016, South Korea [66]	433	7873	0.055	[0.050; 0.060]
Paul, 2016, India [67]	117	6736	0.017	[0.014; 0.020]
**Subtotal**		**73640**	**0.026**	**[0.025; 0.027]**
**Random effects model**			**0.020**	**[0.017; 0.025]**
Heterogeneity: $I^2 = 94\%$, $p < 0.01$				
**Male**				
Bonomi, 2000, Italy [21]	38	1882	0.020	[0.014; 0.027]
Dandona, 2000, India [23]	13	1175	0.011	[0.005; 0.017]
Foster, 2000, Singapore [24]	15	557	0.027	[0.013; 0.040]
Quigley, 2001, USA [25]	33	1851	0.018	[0.012; 0.024]
Weih, 2001, Australia [26**]	40	2210	0.018	[0.013; 0.024]
Bourne, 2003, Thailand [28]	3	296	0.010	[0.000; 0.022]
Jonasson, 2003, Iceland [29**]	22	461	0.048	[0.028; 0.067]
Ramakrishnan , 2003, India [30]	58	2314	0.025	[0.019; 0.031]
Iwase, 2004, Japan [33]	57	1334	0.043	[0.032; 0.054]
Rahman, 2004, Bangladesh [35]	19	1220	0.016	[0.009; 0.023]
Varma, 2004, USA [36**]	139	2555	0.054	[0.046; 0.063]
Nizankowska, 2005, Poland [37]	21	2032	0.010	[0.006; 0.015]
Raychaudhuri, 2005, India [38]	22	629	0.035	[0.021; 0.049]
Vijaya, 2005, India [39]	30	1761	0.017	[0.011; 0.023]
Yamamoto, 2005, Japan [40]	55	1334	0.041	[0.031; 0.052]
He, 2006, China [42]	21	613	0.034	[0.020; 0.049]
Sakata, 2007, Brazil [44]	23	696	0.033	[0.020; 0.046]
Shen, 2008, Singapore [46]	59	1576	0.037	[0.028; 0.047]
Vijaya, 2008, India [47]	64	1710	0.037	[0.028; 0.046]
Garudadri, 2010, India [48]	38	1751	0.022	[0.015; 0.029]
kuzin, 2010, USA [49**]	126	2479	0.051	[0.042; 0.059]
Wang, 2010, China [51**]	63	1909	0.033	[0.025; 0.041]
Kim, 2011, South Korea [53]	28	645	0.043	[0.028; 0.059]
Liang, 2011, China [54]	26	3117	0.008	[0.005; 0.012]
Song, 2011, China [55]	33	2299	0.014	[0.009; 0.019]
Thapa, 2012, Nepal [56]	26	1819	0.014	[0.009; 0.020]
Ashaye, 2013, Nigeria [58]	31	389	0.080	[0.053; 0.107]
Budenz, 2013, Ghana [59]	176	2224	0.079	[0.068; 0.090]
Narayanaswamy, 2013, Singapore [60]	28	1706	0.016	[0.010; 0.022]
Baskaran, 2015, Singapore [63]	41	1662	0.025	[0.017; 0.032]
He, 2015, China [64]	37	1068	0.035	[0.024; 0.046]
kim, 2016, South Korea [66]	232	5958	0.039	[0.034; 0.044]
Paul, 2016, India [67]	134	7356	0.018	[0.015; 0.021]
**Subtotal**		**60588**	**0.029**	**[0.028; 0.030]**
**Random effects model**			**0.027**	**[0.022; 0.032]**
Heterogeneity: $I^2 = 94\%$, $p < 0.01$				
**Fixed effect model**		**134228**	**0.027**	**[0.026; 0.028]**
**Random effects model**			**0.023**	**[0.020; 0.027]**
Heterogeneity: $I^2 = 94\%$, $p < 0.01$				
Residual heterogeneity: $I^2 = 94\%$, $p < 0.01$				

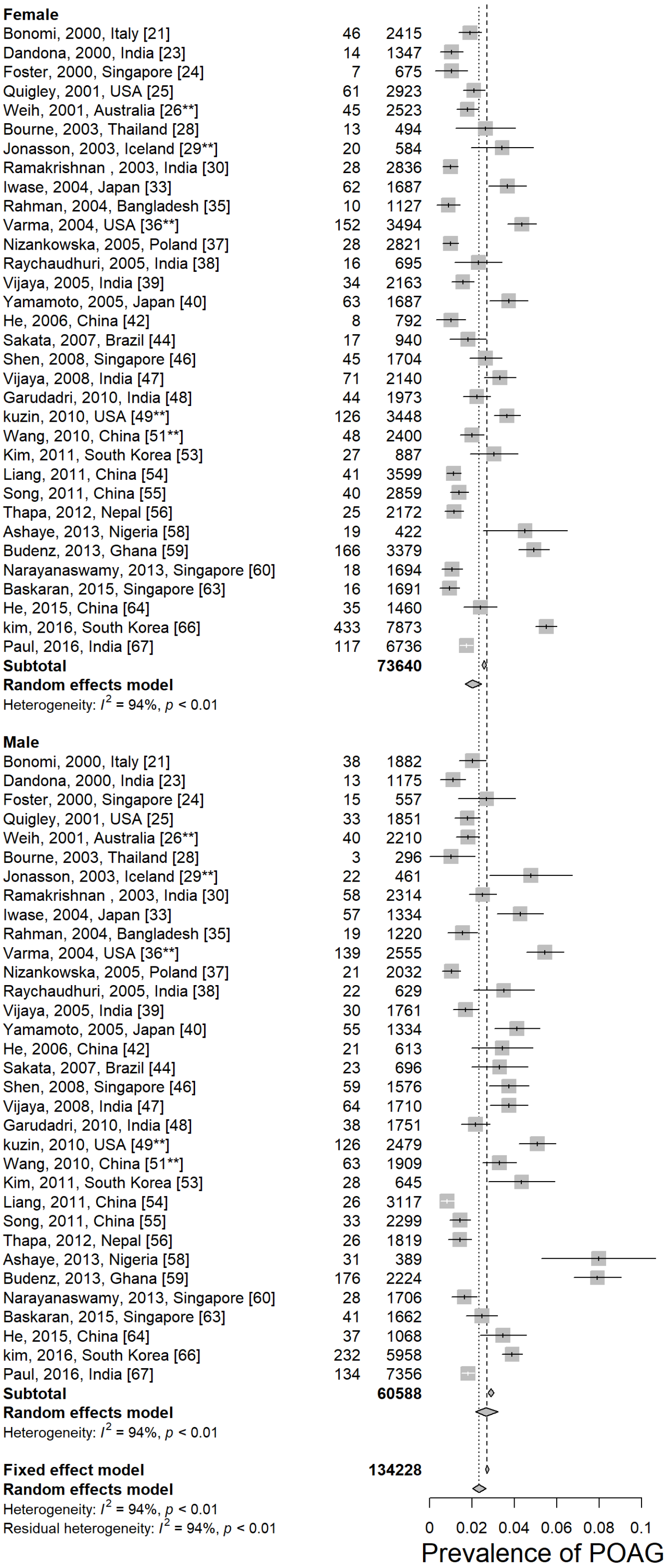

Figure S5. POAG prevalence by gender groups. **: studies that reported prevalence of open-angle glaucoma (OAG), but not POAG.

Study (Group by Age)	Glaucoma	Total	Prevalence	95% CI
**<40**				
Dandona, 2000, India [23]	1	1588	0.001	[0.000; 0.002]
Ntim-Amponsah, 2004, Ghana [34]	28	476	0.059	[0.038; 0.080]
Liang, 2011, China [54]	2	1236	0.002	[0.000; 0.004]
**Subtotal**		**3300**	**0.009**	**[0.007; 0.013]**
**Random effects model**			**0.004**	**[0.000; 0.044]**
Heterogeneity: I^2 = 92%, p < 0.01				
**40-49**				
Dandona, 2000, India [23]	5	395	0.013	[0.002; 0.024]
Quigley, 2001, USA [25]	8	1594	0.005	[0.002; 0.008]
Weih, 2001, Australia [26**]	1	1260	0.001	[0.000; 0.002]
Rotchford, 2002, South Africa [27]	3	254	0.012	[0.000; 0.025]
Ramakrishnan , 2003, India [30]	7	2066	0.003	[0.001; 0.006]
Iwase, 2004, Japan [33]	16	783	0.020	[0.011; 0.030]
Ntim-Amponsah, 2004, Ghana [34]	29	457	0.063	[0.041; 0.086]
Varma, 2004, USA [36**]	31	2363	0.013	[0.009; 0.018]
Vijaya, 2005, India [39]	10	1585	0.006	[0.002; 0.010]
Sakata, 2007, Brazil [44]	7	687	0.010	[0.003; 0.018]
Shen, 2008, Singapore [46]	14	814	0.017	[0.008; 0.026]
Vijaya, 2008, India [47]	32	1419	0.023	[0.015; 0.030]
kuzin, 2010, USA [49**]	29	2337	0.012	[0.008; 0.017]
Wang, 2010, China [51**]	26	1444	0.018	[0.011; 0.025]
Kim, 2011, South Korea [53]	3	211	0.014	[0.000; 0.030]
Liang, 2011, China [54]	4	1325	0.003	[0.000; 0.006]
Thapa, 2012, Nepal [56]	3	1484	0.002	[0.000; 0.004]
Ashaye, 2013, Nigeria [58]	12	259	0.046	[0.021; 0.072]
Budenz, 2013, Ghana [59]	96	2606	0.037	[0.030; 0.044]
Narayanaswamy, 2013, Singapore [60]	11	957	0.011	[0.005; 0.018]
Baskaran, 2015, Singapore [63]	3	705	0.004	[0.000; 0.009]
kim, 2016, South Korea [66]	113	4034	0.028	[0.023; 0.033]
Paul, 2016, India [67]	60	4826	0.012	[0.009; 0.016]
**Subtotal**		**33865**	**0.015**	**[0.014; 0.017]**
**Random effects model**			**0.011**	**[0.008; 0.017]**
Heterogeneity: I^2 = 94%, p < 0.01				
**50-59**				
Dandona, 2000, India [23]	6	260	0.023	[0.005; 0.041]
Quigley, 2001, USA [25]	8	1362	0.006	[0.002; 0.010]
Weih, 2001, Australia [26**]	11	1333	0.008	[0.003; 0.013]
Rotchford, 2002, South Africa [27]	5	269	0.019	[0.002; 0.035]
Jonasson, 2003, Iceland [29**]	2	362	0.006	[0.000; 0.013]
Ramakrishnan , 2003, India [30]	23	1466	0.016	[0.009; 0.022]
Iwase, 2004, Japan [33]	26	959	0.027	[0.017; 0.037]
Ntim-Amponsah, 2004, Ghana [34]	26	369	0.070	[0.044; 0.097]
Varma, 2004, USA [36**]	54	1853	0.029	[0.021; 0.037]
Raychaudhuri, 2005, India [38]	16	637	0.025	[0.013; 0.037]
Vijaya, 2005, India [39]	16	985	0.016	[0.008; 0.024]
He, 2006, China [42]	5	475	0.011	[0.001; 0.020]
Sakata, 2007, Brazil [44]	15	500	0.030	[0.015; 0.045]
Shen, 2008, Singapore [46]	24	957	0.025	[0.015; 0.035]
Vijaya, 2008, India [47]	40	1120	0.036	[0.025; 0.047]
kuzin, 2010, USA [49**]	54	1831	0.029	[0.022; 0.037]
Wang, 2010, China [51**]	19	1188	0.016	[0.009; 0.023]
Kim, 2011, South Korea [53]	7	321	0.022	[0.006; 0.038]
Liang, 2011, China [54]	25	2457	0.010	[0.006; 0.014]
Thapa, 2012, Nepal [56]	9	1094	0.008	[0.003; 0.014]
Ashaye, 2013, Nigeria [58]	12	226	0.053	[0.024; 0.082]
Budenz, 2013, Ghana [59]	100	1587	0.063	[0.051; 0.075]
Narayanaswamy, 2013, Singapore [60]	9	1077	0.008	[0.003; 0.014]
Baskaran, 2015, Singapore [63]	17	1113	0.015	[0.008; 0.022]
He, 2015, China [64]	19	982	0.019	[0.011; 0.028]
kim, 2016, South Korea [66]	173	3751	0.046	[0.039; 0.053]
Paul, 2016, India [67]	67	3902	0.017	[0.013; 0.021]
**Subtotal**		**32436**	**0.024**	**[0.023; 0.026]**
**Random effects model**			**0.020**	**[0.016; 0.026]**
Heterogeneity: I^2 = 91%, p < 0.01				

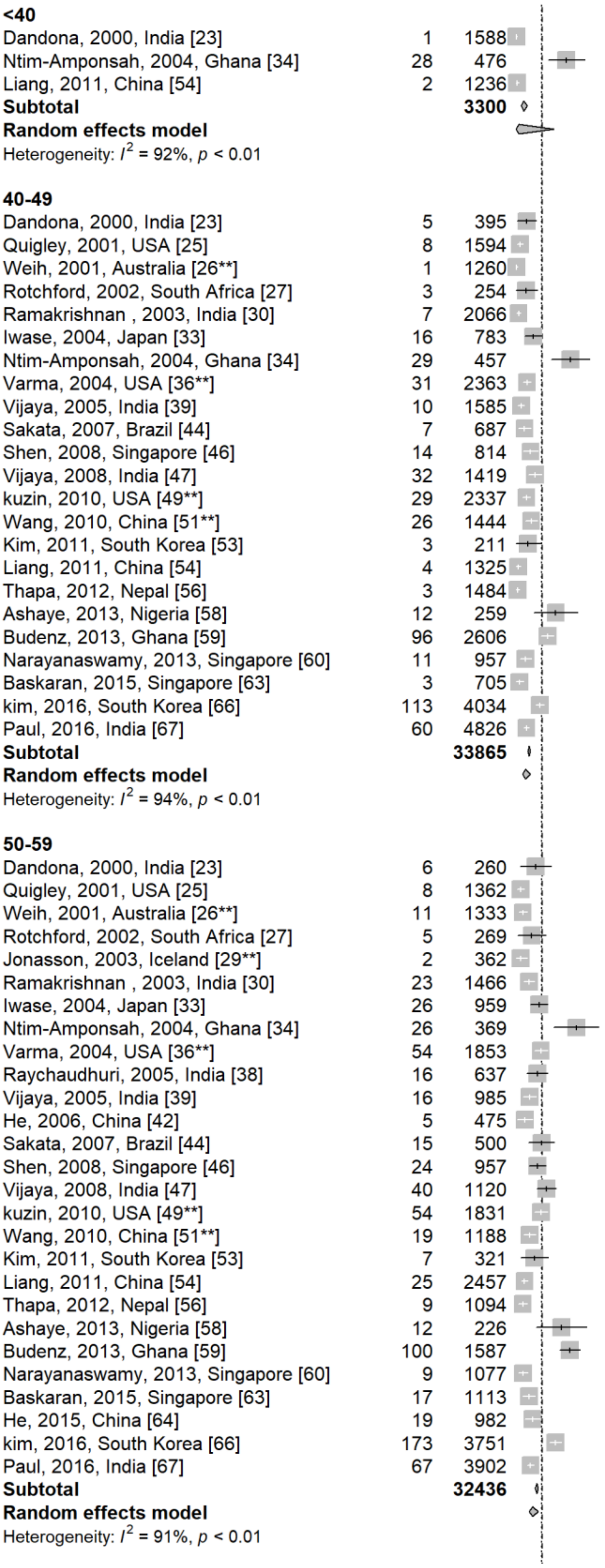

Figure S6. POAG prevalence by age groups. **: studies that reported prevalence of open-angle glaucoma (OAG), but not POAG.

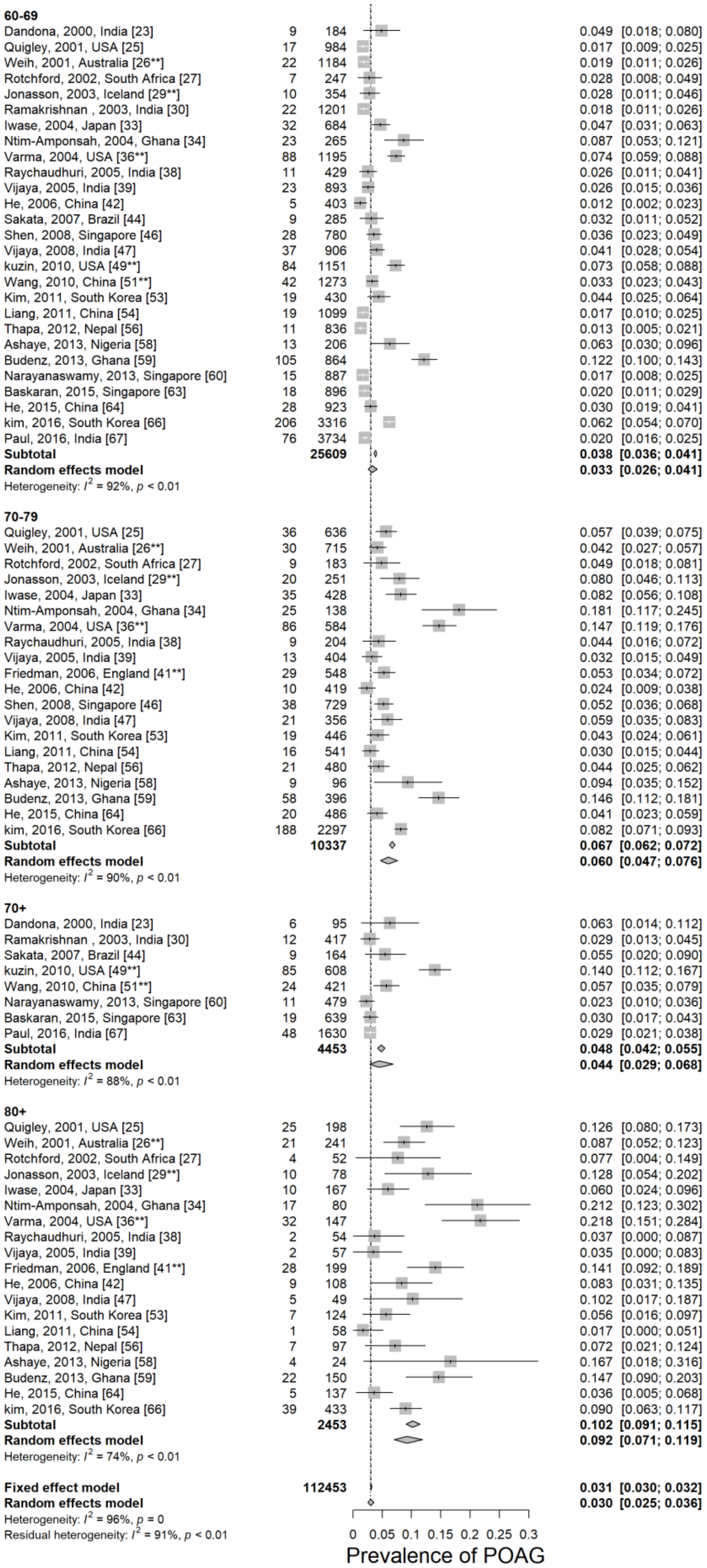

Figure S6. (continued) POAG prevalence by age groups. **: studies that reported prevalence of open-angle glaucoma (OAG), but not POAG.

Study	Glaucoma	Total	Prevalence	95% CI
**Africa**				
Buhrmann, 2000, Tanzania [22]	100	3268	0.031	[0.025; 0.037]
Rotchford, 2002, South Africa [27]	28	1005	0.028	[0.018; 0.038]
Rotchford, 2003, South Africa [31]	31	839	0.037	[0.024; 0.050]
Ntim-Amponsah, 2004, Ghana [34]	148	1785	0.083	[0.070; 0.096]
Ashaye, 2013, Nigeria [58]	50	811	0.062	[0.045; 0.078]
Budenz, 2013, Ghana [59]	381	5603	0.068	[0.061; 0.075]
Kyari, 2015, Nigeria [65]	208	13591	0.015	[0.013; 0.017]
**Subtotal**		**26902**	**0.035**	**[0.033; 0.037]**
**Random effects model**			**0.040**	**[0.026; 0.061]**
Heterogeneity: I^2 = 97%, p < 0.01				
**Asia**				
Dandona, 2000, India [23]	27	2522	0.011	[0.007; 0.015]
Foster, 2000, Singapore [24]	22	1232	0.018	[0.010; 0.025]
Bourne, 2003, Thailand [28]	16	790	0.020	[0.010; 0.030]
Ramakrishnan , 2003, India [30]	64	5150	0.012	[0.009; 0.015]
Iwase, 2004, Japan [33]	119	3021	0.039	[0.032; 0.046]
Rahman, 2004, Bangladesh [35]	29	2347	0.012	[0.008; 0.017]
Raychaudhuri, 2005, India [38]	38	1324	0.029	[0.020; 0.038]
Vijaya, 2005, India [39]	64	3924	0.016	[0.012; 0.020]
Yamamoto, 2005, Japan [40]	118	3021	0.039	[0.032; 0.046]
He, 2006, China [42]	29	1405	0.021	[0.013; 0.028]
Casson, 2007, Myanmar [43]	42	2076	0.020	[0.014; 0.026]
Shen, 2008, Singapore [46]	104	3280	0.032	[0.026; 0.038]
Vijaya, 2008, India [47]	135	3850	0.035	[0.029; 0.041]
Garudadri, 2010, India [48]	82	3724	0.022	[0.017; 0.027]
Sia, 2010, Sri Lanka [50]	32	1375	0.023	[0.015; 0.031]
Wang, 2010, China [51**]	111	4439	0.025	[0.020; 0.030]
Al-Mansouri, 2011, Qatar [52]	44	3149	0.014	[0.010; 0.018]
Kim, 2011, South Korea [53]	55	1532	0.036	[0.027; 0.045]
Liang, 2011, China [54]	67	6716	0.010	[0.008; 0.012]
Song, 2011, China [55]	73	5158	0.014	[0.011; 0.017]
Thapa, 2012, Nepal [56]	51	3991	0.013	[0.009; 0.016]
Zhong, 2012, China [57]	22	2133	0.010	[0.006; 0.015]
Narayanaswamy, 2013, Singapore [60]	46	3400	0.014	[0.010; 0.017]
Pakravan, 2013, Iran [61]	64	2320	0.028	[0.021; 0.034]
Yamamoto, 2014, Japan [62]	152	3762	0.040	[0.034; 0.047]
Baskaran, 2015, Singapore [63]	57	3353	0.017	[0.013; 0.021]
He, 2015, China [64]	72	2528	0.028	[0.022; 0.035]
kim, 2016, South Korea [66]	710	13831	0.051	[0.048; 0.055]
Paul, 2016, India [67]	251	14092	0.018	[0.016; 0.020]
Chassid, 2018, Israel [68]	454	15122	0.030	[0.027; 0.033]
Addepalli, 2019, India [69]	41	3833	0.011	[0.007; 0.014]
**Subtotal**		**128400**	**0.025**	**[0.024; 0.026]**
**Random effects model**			**0.021**	**[0.018; 0.024]**
Heterogeneity: I^2 = 95%, p < 0.01				
**Europe**				
Bonomi, 2000, Italy [21]	84	4297	0.020	[0.015; 0.024]
Jonasson, 2003, Iceland [29**]	42	1045	0.040	[0.028; 0.052]
Anton, 2004, Spain [32]	12	569	0.021	[0.009; 0.033]
Nizankowska, 2005, Poland [37]	49	4853	0.010	[0.007; 0.013]
Friedman, 2006, England [41**]	175	3306	0.053	[0.045; 0.061]
Topouzis, 2007, Greece [45]	69	2554	0.027	[0.021; 0.033]
McCann, 2020, Northern Ireland [70]	48	3221	0.015	[0.011; 0.019]
**Subtotal**		**19845**	**0.024**	**[0.022; 0.026]**
**Random effects model**			**0.023**	**[0.015; 0.035]**
Heterogeneity: I^2 = 95%, p < 0.01				
**N. America**				
Quigley, 2001, USA [25]	94	4774	0.020	[0.016; 0.024]
Varma, 2004, USA [36**]	291	6142	0.047	[0.042; 0.053]
kuzin, 2010, USA [49**]	252	5927	0.043	[0.037; 0.048]
**Subtotal**		**16843**	**0.038**	**[0.035; 0.041]**
**Random effects model**			**0.034**	**[0.022; 0.053]**
Heterogeneity: I^2 = 97%, p < 0.01				
**Oceania**				
Weih, 2001, Australia [26**]	85	4633	0.018	[0.014; 0.022]
**Subtotal**		**4633**	**0.018**	**[0.015; 0.023]**
**Random effects model**			**0.018**	**[0.015; 0.023]**
Heterogeneity: not applicable				
**S. America**				
Sakata, 2007, Brazil [44]	40	1636	0.024	[0.017; 0.032]
**Subtotal**		**1636**	**0.024**	**[0.018; 0.033]**
**Random effects model**			**0.024**	**[0.018; 0.033]**
Heterogeneity: not applicable				
**Fixed effect model**		**198259**	**0.027**	**[0.026; 0.028]**
**Random effects model**			**0.024**	**[0.020; 0.028]**
Heterogeneity: I^2 = 97%, p < 0.01				
Residual heterogeneity: I^2 = 97%, p < 0.01				

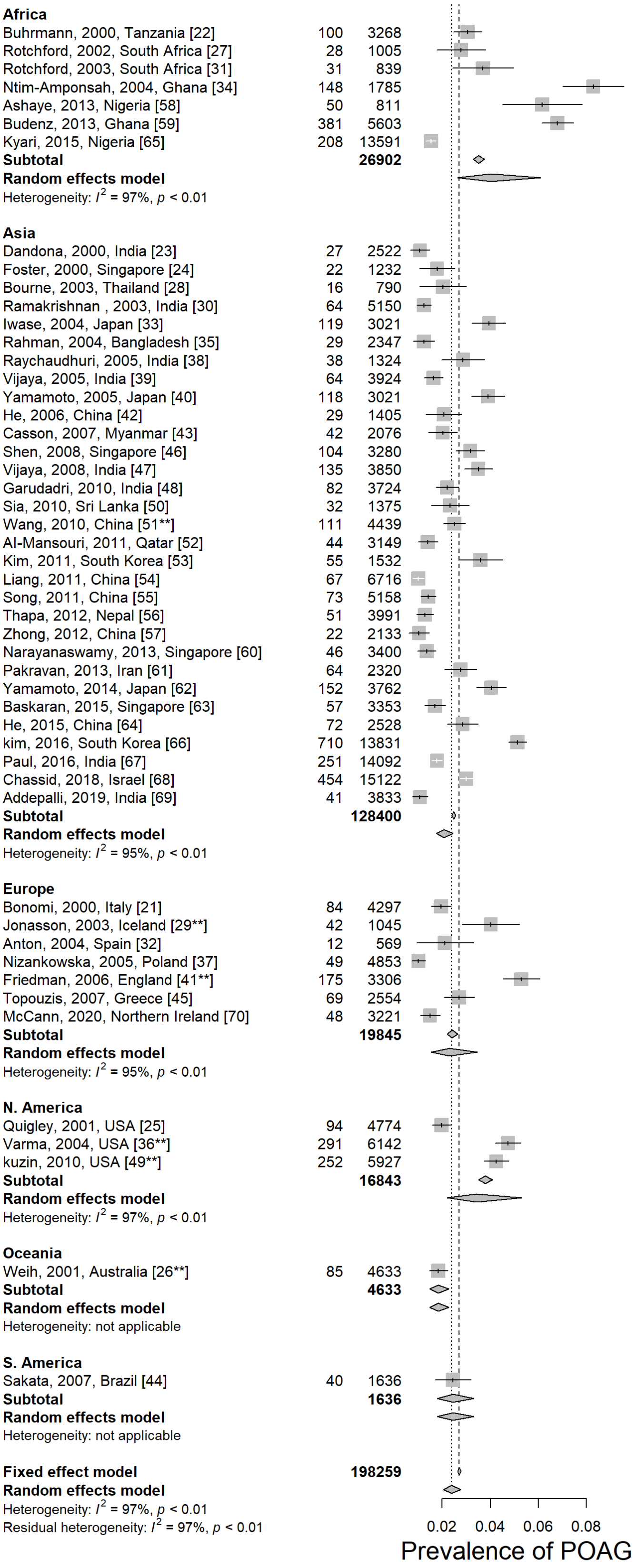

Figure S7. POAG prevalence by continents. **: studies that reported prevalence of open-angle glaucoma (OAG), but not POAG.

Study	Glaucoma	Total	Prevalence	95% CI
**Urban**				
Dandona, 2000, India [23]	27	2522	0.011	[0.007; 0.015]
Weih, 2001, Australia [26**]	56	3264	0.017	[0.013; 0.022]
Bourne, 2003, Thailand [28]	16	790	0.020	[0.010; 0.030]
Rotchford, 2003, South Africa [31]	31	839	0.037	[0.024; 0.050]
He, 2006, China [42]	29	1405	0.021	[0.013; 0.028]
Topouzis, 2007, Greece [45]	69	2554	0.027	[0.021; 0.033]
Shen, 2008, Singapore [46]	104	3280	0.032	[0.026; 0.038]
Vijaya, 2008, India [47]	135	3850	0.035	[0.029; 0.041]
Garudadri, 2010, India [48]	37	934	0.040	[0.027; 0.052]
Budenz, 2013, Ghana [59]	381	5603	0.068	[0.061; 0.075]
Narayanaswamy, 2013, Singapore [60]	46	3400	0.014	[0.010; 0.017]
Baskaran, 2015, Singapore [63]	57	3353	0.017	[0.013; 0.021]
He, 2015, China [64]	72	2528	0.028	[0.022; 0.035]
Paul, 2016, India [67]	150	7128	0.021	[0.018; 0.024]
**Subtotal**		**41450**	**0.029**	**[0.028; 0.031]**
**Random effects model**			**0.025**	**[0.019; 0.032]**
Heterogeneity: I^2 = 94%, p < 0.01				
**Rural**				
Buhrmann, 2000, Tanzania [22]	100	3268	0.031	[0.025; 0.037]
Weih, 2001, Australia [26**]	29	1369	0.021	[0.014; 0.029]
Rotchford, 2002, South Africa [27]	28	1005	0.028	[0.018; 0.038]
Ramakrishnan , 2003, India [30]	64	5150	0.012	[0.009; 0.015]
Raychaudhuri, 2005, India [38]	38	1324	0.029	[0.020; 0.038]
Vijaya, 2005, India [39]	64	3924	0.016	[0.012; 0.020]
Casson, 2007, Myanmar [43]	42	2076	0.020	[0.014; 0.026]
Garudadri, 2010, India [48]	45	2790	0.016	[0.011; 0.021]
Sia, 2010, Sri Lanka [50]	32	1375	0.023	[0.015; 0.031]
Kim, 2011, South Korea [53]	55	1532	0.036	[0.027; 0.045]
Liang, 2011, China [54]	67	6716	0.010	[0.008; 0.012]
Song, 2011, China [55]	73	5158	0.014	[0.011; 0.017]
Zhong, 2012, China [57]	22	2133	0.010	[0.006; 0.015]
Yamamoto, 2014, Japan [62]	152	3762	0.040	[0.034; 0.047]
Paul, 2016, India [67]	101	6964	0.015	[0.012; 0.017]
Addepalli, 2019, India [69]	41	3833	0.011	[0.007; 0.014]
**Subtotal**		**52379**	**0.018**	**[0.017; 0.019]**
**Random effects model**			**0.019**	**[0.015; 0.023]**
Heterogeneity: I^2 = 91%, p < 0.01				
**Mixed or Unknown**				
Bonomi, 2000, Italy [21]	84	4297	0.020	[0.015; 0.024]
Foster, 2000, Singapore [24]	22	1232	0.018	[0.010; 0.025]
Quigley, 2001, USA [25]	94	4774	0.020	[0.016; 0.024]
Weih, 2001, Australia [26**]	85	4633	0.018	[0.014; 0.022]
Jonasson, 2003, Iceland [29**]	42	1045	0.040	[0.028; 0.052]
Anton, 2004, Spain [32]	12	569	0.021	[0.009; 0.033]
Iwase, 2004, Japan [33]	119	3021	0.039	[0.032; 0.046]
Ntim-Amponsah, 2004, Ghana [34]	148	1785	0.083	[0.070; 0.096]
Rahman, 2004, Bangladesh [35]	29	2347	0.012	[0.008; 0.017]
Varma, 2004, USA [36**]	291	6142	0.047	[0.042; 0.053]
Nizankowska, 2005, Poland [37]	49	4853	0.010	[0.007; 0.013]
Yamamoto, 2005, Japan [40]	118	3021	0.039	[0.032; 0.046]
Friedman, 2006, England [41**]	175	3306	0.053	[0.045; 0.061]
Sakata, 2007, Brazil [44]	40	1636	0.024	[0.017; 0.032]
Garudadri, 2010, India [48]	82	3724	0.022	[0.017; 0.027]
kuzin, 2010, USA [49**]	252	5927	0.043	[0.037; 0.048]
Wang, 2010, China [51**]	111	4439	0.025	[0.020; 0.030]
Al-Mansouri, 2011, Qatar [52]	44	3149	0.014	[0.010; 0.018]
Thapa, 2012, Nepal [56]	51	3991	0.013	[0.009; 0.016]
Ashaye, 2013, Nigeria [58]	50	811	0.062	[0.045; 0.078]
Pakravan, 2013, Iran [61]	64	2320	0.028	[0.021; 0.034]
Kyari, 2015, Nigeria [65]	208	13591	0.015	[0.013; 0.017]
kim, 2016, South Korea [66]	710	13831	0.051	[0.048; 0.055]
Paul, 2016, India [67]	251	14092	0.018	[0.016; 0.020]
Chassid, 2018, Israel [68]	454	15122	0.030	[0.027; 0.033]
McCann, 2020, Northern Ireland [70]	48	3221	0.015	[0.011; 0.019]
**Subtotal**		**126879**	**0.029**	**[0.028; 0.030]**
**Random effects model**			**0.026**	**[0.021; 0.032]**
Heterogeneity: I^2 = 98%, p < 0.01				
**Fixed effect model**		**220708**	**0.026**	**[0.026; 0.027]**
**Random effects model**			**0.023**	**[0.020; 0.027]**
Heterogeneity: I^2 = 96%, p = 0				
Residual heterogeneity: I^2 = 97%, p < 0.01				

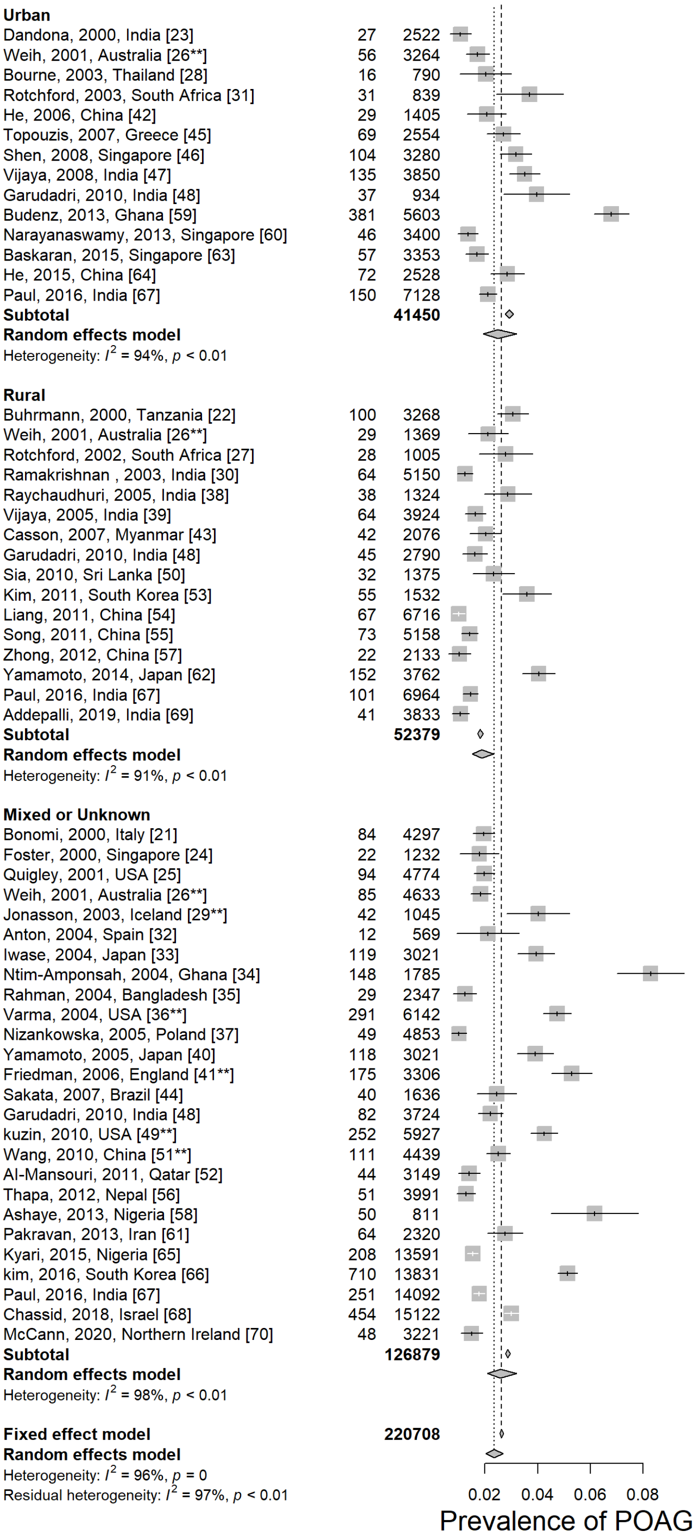

.

Figure S8. POAG prevalence by habitation areas. **: studies that reported prevalence of open-angle glaucoma (OAG), but not POAG.

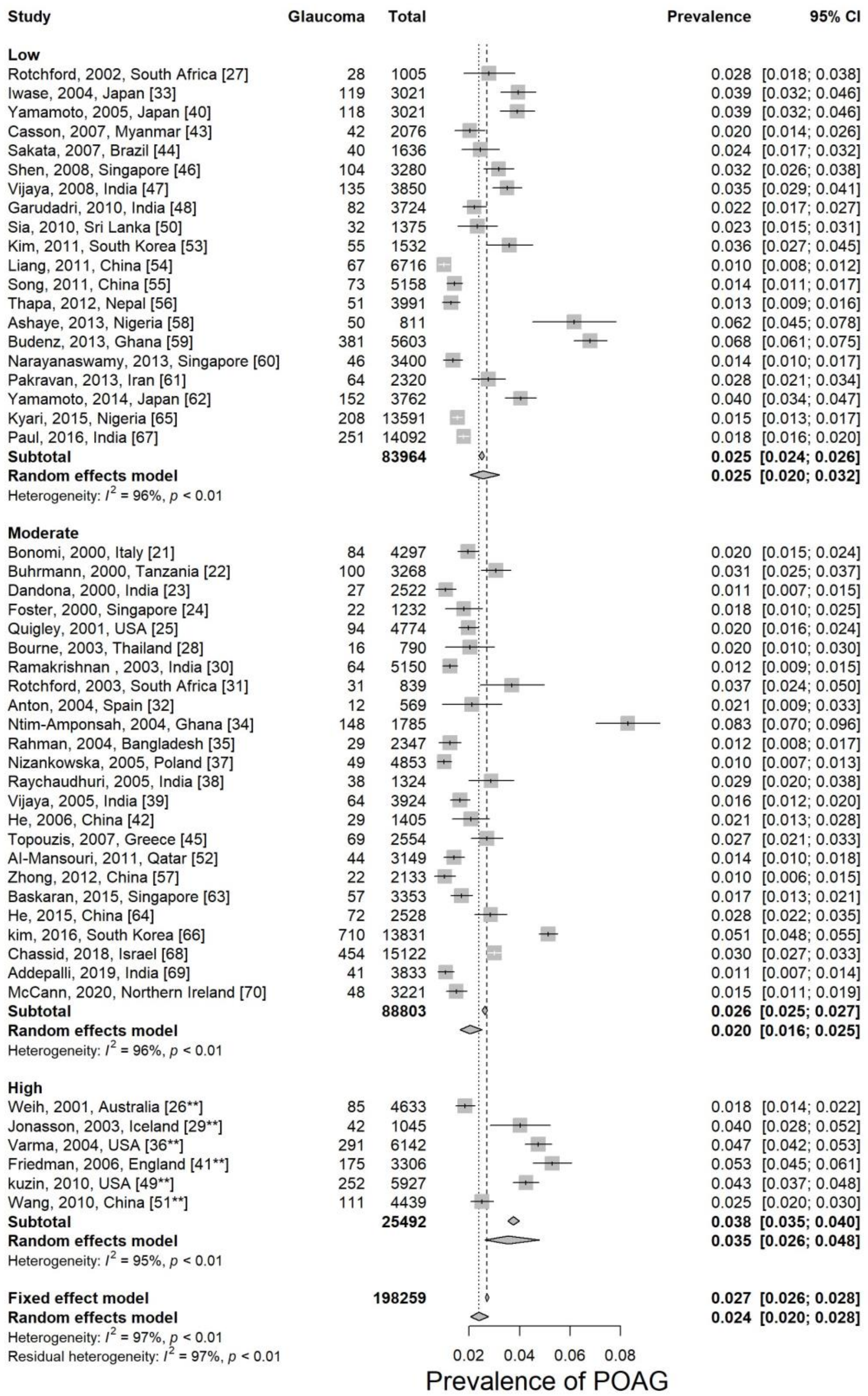

Figure S9. POAG prevalence grouped by risk of bias. **: studies that reported prevalence of open-angle glaucoma (OAG), but not POAG.
